# RNA Sequencing Reveals the Regulation Mechanism of Yunnan Baiyao in Treating Skin Infection Caused by *Staphylococcus aureus*

**DOI:** 10.1155/2022/6348157

**Published:** 2022-10-12

**Authors:** Jiachan Zhang, Changtao Wang, Quan An, Qianghua Quan, Yiming Wang, Tong Huo, Jitao Liu

**Affiliations:** ^1^Beijing Key Lab of Plant Resource Research and Development, College of Chemistry and Materials engineering, Beijing Technology and Business University, No. 33 Fucheng Road, Haidian District, Beijing 100048, China; ^2^East Asia Skin Health Research Center, Fucheng Road, Haidian District, Beijing 100048, China; ^3^Institute of Cosmetic Regulatory Science, Beijing Technology and Business University, Fucheng Road, Haidian District, Beijing 100048, China; ^4^Yunnan Baiyao Group Co Ltd, Kunming, China

## Abstract

Yunnan Baiyao is a well-known traditional Chinese medicine that can be formulated into a powder or capsule form. The mechanism by which it exerts its anti-inflammation effect, which is used in skin care products, needs to be further explored. In this study, we established the *Staphylococcus aureus*-induced mouse skin inflammatory model to investigate the effects of Yunnan Baiyao by the method of RNA-sequencing technology. The mice were randomly assigned to three groups, and those were control, model, and the Yunnan Baiyao-treated (YNtreated) group. Key genes and pathways were identified using bioinformatics analyses. In the study, we obtained 1,053 differentially expressed genes (DEGs) induced by Yunnan Baiyao. The 233 upregulated genes were enriched in 32 GO terms and 5 KEGG pathways, focused on the items, such as wound healing, cell metabolism, and proliferation, indicating the accelerating effects of Yunnan Baiyao on these aspects. The 820 downregulated genes were enriched mainly in the items, including the regulation of inflammation factor production, immune responses, and regulation of structure dermal components. Besides, Yunnan Baiyao reversed the expressions of 277 (201 decreased and 76 increased DEGs, respectively) induced by *S. aureus*. Ten key regulatory nodes (MMP2, PLK1, CCNB1, TLR4, CDK1, CCNA2, CDC25C, PDGFRA, MYOC, and KNG1) were identified by the construction of the protein interaction network, half of which were related to cell proliferation. VAV1 was another hub node that was affected by Yunnan Baiyao (Top 20). In the study, VAV1 and TLR4 can be considered key module genes in inflammation regulation. In conclusion, this study found that Yunnan Baiyao can significantly relieve inflammatory symptoms by regulating genes and pathways involved in the regulation of inflammation and immune response and also helped to deepen our understanding of the associated molecular mechanisms.

## 1. Introduction

Skin inflammation is a relatively common skin condition that is significantly associated with recurrent skin infections, disease severity, and *Staphylococcus aureus* (*S. aureus*) colonization [1–3]. *S. aureus* proteins, *δ*-toxin that stimulates mast cells, *α*-toxin that damages keratinocytes, phenol-soluble molecules that stimulate cytokine release by keratinocytes, protein A that triggers inflammatory responses from keratinocytes, superantigens that trigger B cell expansion and cytokine release, and proinflammatory lipoproteins that contribute to the intensity of symptoms [4], may be important for inflammation. Thus, it can be seen that *S. aureus* is a key component responsible for inflammation and a particularly troublesome microorganism in the field of dermatology.

The rapid evolution of whole transcriptome profiling using next-generation high-throughput RNA-sequencing (RNA-Seq) has shed light on the complex mechanisms and pathways involved in skin inflammation [5]. Gene expression profiling of RNAs isolated from skin biopsy specimens has revealed significant differences in the expression profiles of patients with skin inflammation, compared with those without, implicating type 2 immune pathways, as well as dysregulation of the skin barrier, through modification of cornification genes and lipid biosynthesis [6, 7]. Although many drugs have been used to alleviate skin inflammation [8, 9], changes in skin genome expression caused by drug treatment need to be studied further. Therefore, there is an urgent need to identify specific treatment response groups so that approval for these drugs can be obtained as soon as possible.

Yunnan Baiyao, originally called Qu-huan-zhang-wang-ying-bai-bao, is a well-known traditional Chinese medicine formulation that is administered in powder or capsule form [10–17], although its exact components are unknown. It exhibits procoagulant, wound healing, anti-inflammatory, analgesic, antibacterial, antitumor, and other effects [18, 19] and is used to cure bleeding, tissue injuries, and inflammation-related or infection-related skin diseases. Nevertheless, the mechanisms underlying the pharmacological effects of Yunnan Baiyao have not been elucidated.

A clinical study conducted in 2019 showed that Yunnan Baiyao can reduce hospital-acquired pressure ulcers caused due to *S. aureus* infection by suppressing virulence gene expression and biofilm formation [16]. However, the pharmacological assessment is still lacking. In this study, we established a mouse model of intradermal *S. aureus* infection and analyzed the local response in the untreated and Yunnan Baiyao-treated animals.

The transcriptomic profiles of the skin tissues were analyzed and functionally annotated in order to identify critical pathways and pivotal related genes that may be involved in the anti-inflammatory effects of Yunnan Baiyao.

## 2. Materials and Methods

### 2.1. Materials

The strain of *Staphylococcus aureus*S1 was purchased from China General Microbiological Culture Collection Center (CGMCC Number: 1.4519), and its identity was confirmed to be *Staphylococcus aureus* (ATCC Number: 25923). Before use, *S. aureus* bacteria were streaked onto a nutrient agar (NA) plate and grown overnight at 37°C in a bacterial incubator. Single colonies were picked and cultured in a nutrient broth medium at 37°C in a shaking incubator (200 rpm) overnight (15 h), followed by a 2% subculture at 37°C for 2 h to obtain midlogarithmic phase bacteria. The bacteria were harvested through centrifugation and washed and resuspended in normal saline solution at the concentration of 3 × 10^7^ CFU/100 *μ*L [20]. The absorbance at 600 nm was determined to estimate the number of CFU, which was verified through overnight culture on an NA plate.

### 2.2. Animals and Treatment

Fifteen Balb/c-Nu nude mice (6–8 weeks, male) with an average weight of 20 g were purchased from Beijing Vital River Laboratory Animal Technology Co., Ltd. (Beijing, China). 2, 2, 2,-tribromoethanol (98.5%) was purchased from J&K Scientific Ltd. The animal experimental procedures described in the following section were approved by the Ethics Committee of the College of Chemistry and Materials Engineering, Beijing Technology and Business University and were performed in accordance with the relevant guidelines and regulations on the use of laboratory animals at the College of Chemistry and Materials Engineering, Beijing Technology and Business University.

The mouse model of intradermal *S. aureus* infection was established as previously described [21]. In brief, the nude mice were housed under standard conditions and adaptively fed for a week with standard laboratory chow and then randomly divided into the control, model, and YN-treated groups (*n* = 5). The mice in the model and YN-treated groups were each injected subcutaneously with a 100 *μ*L of 3 × 10^7^ CFU *S. aureus* into their right hind limbs.

The control mice received the same volume of normal saline. All mice had free access to food and water. Inflammation was observed the day after injection. The inflamed skin area was topically treated with 100 *μ*L of 1 mg/mL of Yunnan Baiyao (Yunnan Baiyao Group Co., Ltd. Kunming, China), twice daily for three days. The treatment duration with YB was set at 3 days based on published literature [22–24]. The mice in the model group were treated with the same dose of phosphate buffered saline (PBS).

Three mice from each group were euthanized with an intraperitoneal injection of tribromoethanol (240 mg/kg BW), and the inflamed skin was peeled off. The tissue samples from each mouse were divided into two parts, one for paraffin embedding and hematoxylin-eosin (HE) staining and the other for RNA isolation and transcriptomic analysis. HE-stained sections were observed under a microscope, and the thicknesses of the stratum spinosum and stratum epidermis were measured using the image analysis software program CaseViewer (3DHISTECH. Ltd).

### 2.3. RNA Isolation and Sequencing Analysis

Total RNA was extracted using TRIzol® reagent (Sigma Technologies, St Louis, USA) according to the manufacturer's instructions. Only high-quality RNA samples (OD260/280 = 1.8∼2.2, OD260/230 ≥ 2.0, RIN ≥ 6.5, 28S : 18S ≥ 1.0, > 2 *μ*g) were applied for further sequencing with the usage of the TruSeq™ RNA sample preparation kit (Illumina, San Diego, CA, USA) according to the manufacturer's instructions. The critical information about read mapping, bioinformatics tools, and criteria were based on the literature published before [25]. The data were analyzed on the free online platform of Majorbio Cloud Platform (https://www.majorbio.com). RSEM (https://deweylab.biostat.wisc.edu/rsem/) was used to quantify gene abundances [26]. Statistical analysis of DEGs was conducted using the DESeq2 package.  Table S1 represented the trimming and read mapping results of the sequences generated from the skin samples. A *P* value and the fold change (FC) for each gene were calculated to denote its expression difference between libraries. The remaining cDNA samples were returned to the laboratory.

### 2.4. Differentially Expressed Gene Analysis

The differentially expressed genes (DEGs) in the model group compared to the control group and in the YN-treated group compared to the model group were both screened using *P* value < 0.01 and |log_2_FC| > 3 as the criteria. For convenience, we defined model DEGs as the differentially expressed genes in the model group compared with the control and YN-treated DEGs as the differentially expressed genes in the YN-treated group compared with the model group. A Venn diagram was drawn to visualize the overlap between the DEGs of both pairs, and two overlapping groups (Mup vs YNdown and Mdown vs YNup) were identified, which attracted our attention.

The Database for Annotation, Visualization and Integrated Discovery (DAVID version 6.8, https://david.ncifcrf.gov/) was used to identify enriched gene ontology (GO) terms and KEGG pathways among the DEGs [27, 28]. The GO categories included biological processes (PB), molecular functions (MF), and cellular components (CC). The GO clustering analyses of upregulated and downregulated genes were performed with an enrichment score >2 as the criteria. The KEGG database was used to systematically analyze gene functions and pathways. *P* value < 0.05 was the threshold for statistical significance.

### 2.5. Construction of the Protein-Protein Interaction Network

To further investigate the molecular mechanism by which *S. aureus* induced skin inflammation during development and progression, the protein-protein interaction network (PPI) was analyzed using Cytoscape ver. 3.5.1. Weighted protein-protein interactions of human beings were downloaded from the HPRD (Human Protein Reference Database, https://www.hprd.org/, Release 8). HPRD contains curated proteomic information on human proteins [29, 30].

The DEGs were converted from the mouse to the human using the online tool, HCOP: orthology prediction search in the HGNC database (https://www.genenames.org/tools/hcop/). HGNC is responsible for approving unique symbols and names for human loci, including protein coding genes, ncRNA genes, and pseudogenes, to allow unambiguous scientific communication [31–33].

### 2.6. Quantitative RT-PCR

The RNA was reverse transcribed using the FastQuant RT kit (with gDNase, TIANGEN Biotech, Beijing, China) in a 20 *μ*L reaction volume according to the manufacturer's instructions. QRT-PCR for MMP2, TLR4, CDK1, VAV1, and glyceraldehyde-3-phosphate dehydrogenase (GAPDH) was performed using SuperReal PreMix Plus (SYBR Green, TIANGEN Biotech, Beijing, China) and gene-specific primers (Table S6) on AmpliTaq Gold (PerkinElmer, USA).

All reactions were run in triplicates, and GAPDH was used as the internal control. The cyclic parameters used were predenaturation at 94°C for 30 s, followed by the PCR reaction (45 cycles of 94°C for 15 s, 60°C for 15 s, and 72°C for 10 s), while fluorescence data were collected at 72°C. The relative gene expression levels were analyzed using the 2^−ΔΔCT^ method.

### 2.7. Data Availability

All raw sequencing reads have been submitted to the NCBI Sequence Read Archive (https://www.ncbi.nlm.nih.gov/sra) under the BioProject accession number PRJNA836085.

### 2.8. Statistical Analysis

Statistical analyses were performed using GraphPad Prism software version 7.0. Data for multiple comparisons were compared using two-way ANOVA, as indicated in the figure legends. All data were presented as the mean value where error bars indicated standard deviation (SD). The two-tailed Student's test was used to compare two groups, and *P* < 0.05 was considered statistically significant.

### 2.9. Approval

The Guide for the Care and Use of Laboratory Animals (National Institutes of Health, USA) was closely adhered to in the formulation of all animal experimental procedures. All experimental protocols were approved by the College of Chemistry and Materials Engineering, Beijing Technology and Business University.

## 3. Results and Discussion

Most synthetic anti-inflammatory drugs have considerable adverse effects, which limit their clinical applications [34, 35]. Therefore, efforts have been made to identify novel anti-inflammatory therapies. The Yunnan Baiyao formulation was first developed in Yunnan in 1902 to treat bleeding, ulcer, and infectious diseases [10, 12, 13, 36–40].

### 3.1. Histological Evaluation of the Skin

**Figure 1:**
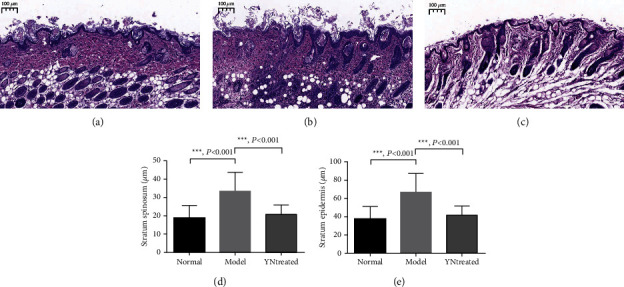
Histological evaluation of the skin (*n* = 5 mice for each group). (a), (b), and (c) representative images of hematoxylin-eosin staining performed to observe the morphological structure of the skin in the control group (a), model group (b), and YN-treated group (c). (d) and (e) were thickness quantifications of the stratum spinosum and stratum epidermis from (a), (b), and (c) (*n* = 5). *P* value from *T*-test. ^*∗*^*P* value < 0.05. Error bars indicate the mean ± SD.

The innate immune response against *S. aureus* infection involves neutrophil recruitment and abscess formation and subsequent inflammation, which results in abscess formation, a hallmark of *S. aureus* infection. Histological examination of the skin samples from the model group revealed a thickened granular layer and the stratum spinosum in the infected area. Furthermore, the stratum epidermis was uneven in thickness, the junction between the epidermis and the dermis was broken, and the hair follicle and sebaceous gland were also fragmented compared to those in the control group (Figures 1(a) and 1(b)). In addition, there was considerable neutrophil infiltration in the dermis and subcutaneous tissues of the infected skin (Figure 1(b)). In the YN-treated group, the epidermal tissue structure was more intact, and the epidermal cells were arranged neatly, with obvious rete ridges and dermal papillae. Wavy fibrous tissue was visible in the dermis, and the hair follicles and sebaceous glands were complete, dense, and evenly distributed. Furthermore, there was no obvious infiltration of inflammatory cells (Figure 1(c)). Quantitative analysis of the thicknesses of the stratum spinosum and epidermis in the different groups showed significant differences between the model group and the control and the YN-treated skin (*P* < 0.001) for both (Figures 1(d) and 1(e)). Taken together, YB alleviated skin inflammation caused by *S. aureus* infection.

### 3.2. Identification of DEGs Related to S. aureus Infection and YB Treatment

**Figure 2:**
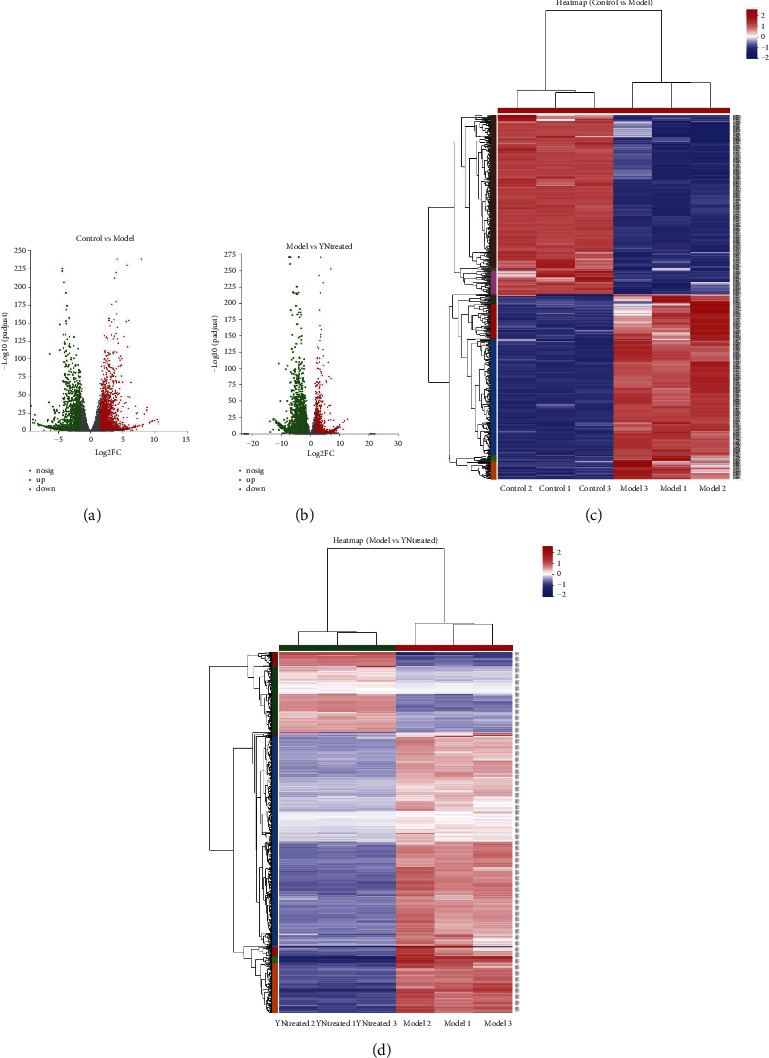
Volcano plots of DEGs (a) control vs model; (b) model vs YN-treated and heatmaps of DEGs (c) control vs model; (d) model vs YN-treated. (a) and (b) red: DEGs with |logFC|>3 in upregulation; green: DEGs with |logFC|>3 in downregulation. (c) and (d) red: upregulation; blue: downregulation.

The DEGs between the control and inflamed skin samples were screened using *P* value< 0.01 and |log_2_FC| > 3 as the thresholds. As shown in the volcano plot in Figure 2(a), 638 genes were differentially expressed in the inflamed skin relative to the control, including 324 upregulated and 314 downregulated genes. The heat map of the model DEGs is shown in Figure 2(c). The transcriptomic analysis of the *S. aureus* infection model was published while this manuscript was in preparation [25]. In brief, 38% of the top 100 upregulated genes in the model group were related to the immune response; apart from eight genes with unknown function, some are involved in encoding metallopeptidases and redox proteins. The top 100 downregulated genes were mainly related to metabolism and cell proliferation. In addition, 16% of the DEGs encoding dermal proteins and nine genes involved in DNA repair were downregulated by *S. aureus* infection. A clear, brief summary was prepared in the supplemental file.

There were 1053 DEGs in the YB-treated group relative to the model group, of which 233 were upregulated and 820 were downregulated (Figure 2(b)). The heat map is shown in Figure 2(d).

### 3.3. GO and KEGG Pathway Analysis of the YN-Treated DEGs

**Figure 3:**
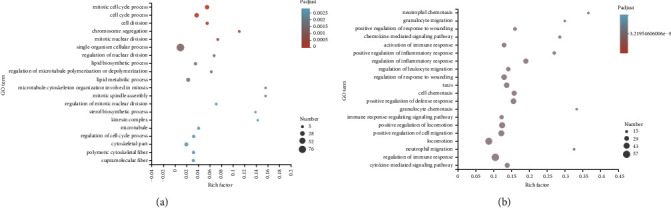
The top 20 enriched GO terms of different expressed genes enrichment between the model group and the YN-treated group in RNA-sequencing. (a) The top 20 upregulated ranging GO terms of the differentially expressed genes between the model group and the YN-treated group. (b) The top 20 downregulated ranging GO terms of the differentially expressed genes between the model group and the YN-treated group. Rich factor: the ratio of the number of target genes divided by the number of all the genes in each pathway. The color of each pot represents the significance of each GO term. The size of the pot represents the number of genes.

**Figure 4:**
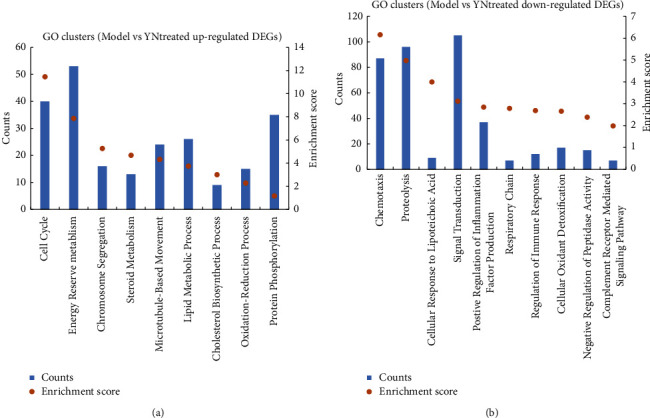
GO clustering results of DEGs between the model group and the YN-treated group (enrichment score >1). (a) The upregulated GO clustering results of DEGs. (b) The downregulated GO clustering results of DEGs.

The top 20 GO terms associated with the YN-treated DEGs are shown in Figure 3 and are arranged in ascending order of the adjusted *P* value. The genes involved in these top 20 enriched GO terms are shown in Table 1. As shown in Figure 3(a), the upregulated genes were significantly enriched in GO terms, including cell cycle processes, cell division, and chromosome segregation, suggesting that some of these DEGs may promote cell proliferation by regulating metabolism and the cell cycle. GO clustering analysis of these DEGs on the basis of the enrichment score (ES) > 1 and gene counts (GC) showed consistent results. As shown in Figure 4(a), the upregulated genes were associated with the cell cycle (ES = 11.47, GC = 38), energy reserve metabolism (ES = 7.86, GC = 53), chromosome segregation (ES = 5.26, GC = 18), steroid metabolism (ES = 4.66, GC = 16), microtubule-based movement (ES = 4.32, GC = 25), the lipid metabolic process (ES = 3.75, GC = 27), the cholesterol biosynthetic process (ES = 3, GC = 10), the oxidation-reduction process (ES = 2.29, GC = 17), and protein phosphorylation (ES = 1.17, GC = 36).

Furthermore, genes related to the single-organism cellular process, wound healing genes, such as growth-related factors (e.g., fibroblast growth factor 6, Fgf6), and the genes involved in chloride channels (Clcn1, Clca3b, and Clca1) and potassium ion fluxes (Kcna7, Kcne1l, Kcnj16, and Kcnmb4os2), were increased in the YN-treated group (Table 1). This is consistent with the findings of Sun that wound healing effects of Yunnan Baiyao are partly mediated by the decrease in bioelectricity at the edge of the wound [41]. Thus, the role of ion fluxes in Yunnan Baiyao-induced wound healing deserves further study.

The downregulated genes were significantly enriched in GO terms associated with immune response, such as neutrophil chemotaxis, the chemokine-mediated signaling pathway, regulation of leukocyte migration, and regulation of inflammatory response, which verified the anti-inflammatory effects of Yunnan Baiyao (Figure 3(b)). According to GO clustering analysis (Figure 4(b)), chemotaxis had the highest enrichment score and gene count (ES = 6.61, GC = 88). Furthermore, positive regulation of inflammation factor production (ES = 2.85, GC = 43), regulation of immune response (ES = 2.69, GC = 12), and the complement receptor-mediated signaling pathway (ES = 1.99, GC = 4) were also clustered, which further underscored the anti-inflammatory effect of Yunnan Baiyao observed in our study. Ren. et al. showed that Yunnan Baiyao treatment significantly decreased IL-1*β*, IL-6, TNF*α*, COX-1, COX-2, and other inflammation-related genes induced by lipopolysaccharide (LPS) [42]. In addition, Li et al. confirmed the immunosuppressive activity of Yunnan Baiyao in a mouse model of colitis, which was mediated by the downregulation of tumor necrosis factor *α* (TNF*α*) and interferon (IFN) *γ* expression in the colonic mucosa and plasma [43]. In our study as well, several TNF*α*-associated genes were inhibited in the YN-treated group, including Tnfaip8l2, Tnfaip6, Tnfsf14, and Tnfsf13b (Table 1). Thus, inhibition of TNF*α* is a possible mechanism underlying the anti-inflammatory effect of Yunnan Baiyao.

The phospholipase A2 (PLA2)/arachidonic acid pathway is a pivotal regulator of inflammatory responses. Arachidonic acid is released from the membrane phospholipids by PLA2 and acts as a substrate for cyclooxygenase-2 (COX-2) and 5-lipoxygenase (5-LOX) in the biosynthesis of eicosanoids (such as prostaglandins and leukotrienes). The inhibition of COXs and LOXs can modulate arachidonic acid metabolism and inhibit inflammation. In fact, several DEGs identified in the YN-treated group are associated with the metabolism of arachidonic acid, although not all of them were among the top 20 GO terms. In particular, arachidonate 5-lipoxygenase activating protein (Alox5ap), arachidonate 5-lipoxygenase (Alox5), phospholipase A2, group VII (Pla2g7), and phospholipase A2, group V (Pla2g5) were downregulated, while arachidonate lipoxygenase (Alox12e) and aldo-keto reductase family 1, member C18 (Akr1c18) were upregulated by Yunnan Baiyao. Likewise, Ren et al. previously showed that the anti-inflammatory effects of Yunnan Baiyao are mediated via the modulation of COX and LOX pathways of arachidonic acid metabolism [11]. Meanwhile, prostaglandin (PG)-associated factors, such as PG-endoperoxide synthase 2, PGI receptor (PGIR), and PGER2, were also repressed by Yunnan Baiyao, which is consistent with the previous reports [44]. He et al. had shown that Yunnan Baiyao ameliorated paw swelling and inflammation in mice by decreasing PGE2 and interleukin 1*β* levels [44].

The toll-like receptors (TLRs) are pattern recognition receptors that initiate innate immune responses to pathogenic infections. Several TLRs recognize *S. aureus* lipopeptides and lipoteichoic acid (LTA), which then triggers secretion of proinflammatory mediators. In this study, several TLRs and pathway intermediates, such as TLR4, TLR7, TLR8, cathepsin K (CTSK), and LPS binding protein (LBP), were upregulated in the inflamed skin. As shown in Table 1, Yunnan Baiyao treatment downregulated multiple TLRs (such as TLR9, TLR8, TLR7, TLR4, TLR2, TLR13, TLR11, and TLR1), which could be the basis of its anti-inflammatory effects.

GO clustering analysis (Figure 4(b)) identified cellular response to lipoteichoic acid (ES = 4.01, GC = 8), which was consistent with the above results. LTA, a unique component of the cell wall of Gram-positive bacteria, plays an important role in the pathogenesis of *S. aureus* [45–47]. Thus, Yunnan Baiyao may alleviate the effects caused by LTA. Matrix metallopeptidases (MMPs) degrade the extracellular matrix, which is critical for pathogenic invasion. Low transcript levels of MMPs were detected in the YN-treated group, which was consistent with the increase in aquaporin 4 (Aqp4), keratin 2, and keratin 9 (Krt2 and Krt9) levels. In addition, proteolysis (ES = 4.98, GC = 97), signal transduction (ES = 3.12, GC = 105), respiratory chain (ES = 2.79, GC = 5), cellular oxidant detoxification (ES = 2.67, GC = 18), and negative regulation of peptidase activity (ES = 2.39, GC = 16) were also upregulated by Yunnan Baiyao (Figure 4(b)).

The KEGG pathways associated with the YN-treated DEGs are shown in Figure S1. The upregulated genes were significantly enriched in terpenoid backbone biosynthesis, steroid biosynthesis, and the cell cycle, and the downregulated genes were significantly enriched in inflammation-related pathways, such as the chemokine signaling pathway, cytokine-cytokine receptor interaction, complement and coagulation cascades, and the IL-17 signaling pathway. Inflammatory responses involving IL-17 likely contribute to arthritis, asthma, skin immune reactions, and autoimmune disorders. Genes participating in the IL-17 signaling pathway, including Lcn2, Cxcl5, S100a8, and S100a8 [25], were upregulated in the model group. As shown in Figure S1(b), Yunnan Baiyao can reduce inflammation partly by suppressing the IL-17 signaling pathway.

### 3.4. Analysis of the Model-Related DEGs Affected by YB Treatment

**Figure 5:**
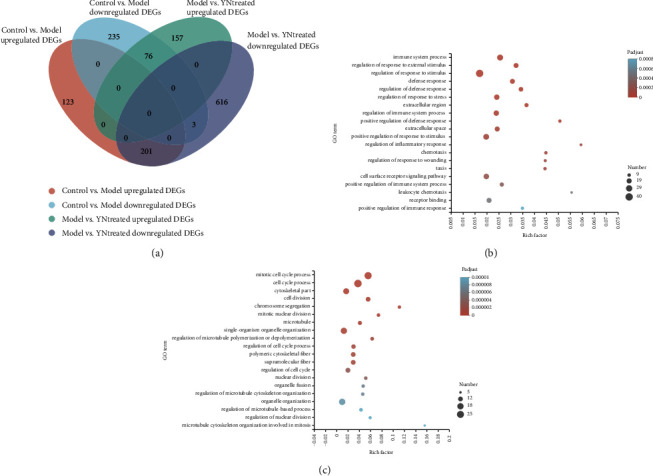
Venn and GO enrichment analysis for Yunnan Baiyao changed DEGs. (a) Venn diagram. (b) GO term enrichment analysis of Mup vs YNdown. (c) GO term enrichment analysis of Mdown vs YNup. Rich factor: the ratio of the number of target genes divided by the number of all the genes in each pathway. The color of each pot represents the significance of each GO term. The size of the pot represents the number of genes.

The Venn diagram in Figure 5(a) shows the overlapping DEGs of the model and YN-treated groups. A total of 201 genes that were upregulated in the model group relative to the control were suppressed in the YN-treated animals and were designated as Mup-YNdown (Figure 5(a)). Furthermore, 72 of these Mup-YNdown genes encode immune response-related proteins, of which eighteen are involved in the immune system process, nine (S100a8, S100a9, Amica1, Ccl6, Ccl9, Cxcr2, C5ar1, ITGAM, and Trem1) are related to neutrophil chemotaxis, seven (Cd163, Hp, Lbp, Serpina3a, Saa1, Saa2, and Saa3) are involved in acute-phase response, and five (Oas1a, Oas1f, Oas1g, Tlr7, and Tlr8) are involved in defense response to virus. Forty-nine genes with the largest decrease in transcript levels encode proteins with known functions in metabolism and cell proliferation, while 16 are functionally uncharacterized. In addition, eight genes encoded enzymes showing metallopeptidase activities, including Cpa3, Adamts15, Mmp11, Ace, LVRN, Mmp2, Dpep1, and Mmp8 (Table S2).

Seventy-six genes that were downregulated in the model group were activated upon YB treatment (Figure 5(a)) and were designated as Mdown-YNup. As shown in Table S3, 26 Mdown-YNup genes encode proteins with known functions in metabolism and cell proliferation, of which eight (Cdc20, Ccna2, Ccnb2, Ccnb1, Plk1, Cdk1, Cdc25c, and Bub1) are involved in cell cycle and cell division, seven genes (Pif1, Neil3, PARPBP, Foxm1, Hist1h3g, Rad54b, and Hist1h2ag) encode proteins involved in DNA repair, and 19 genes encode proteins essential for transport and cell morphogenesis. The kinesin family members, including Kif4, Kif22, Kif20a, Kif14, Kif18b, and Kifc5b, were repressed in the model group and upregulated by Yunnan Baiyao. The kinesin superfamily proteins (KIFs) transport organelles, protein complexes, and mRNAs to specific destinations in a microtubule-dependent and ATP-dependent manner [48], which correlates with the proproliferative effect of Yunnan Baiyao. Three genes were downregulated in both the model and YN-treated groups (Figure 5(a)), including Tgm6, Dio3, and Gprc5. These Mdown-YNdown genes are likely inhibited by Yunnan Baiyao during inflammation.

**Figure 6:**
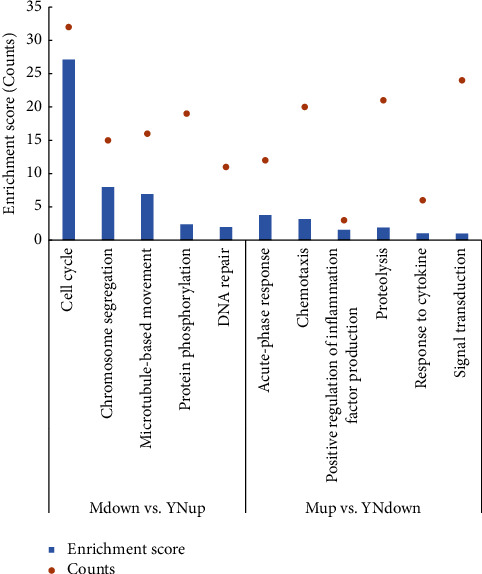
GO clustering results of Yunnan Baiyao changed DEGs (Mdown vs. YNup, Mup vs. YNdown, and the enrichment score>1).

GO enrichment analysis showed that the Mup-YNdown genes were most significantly enriched in terms associated with the regulation of inflammatory response, leucocyte chemotaxis, positive regulation of defense response, and positive regulation of immune response (Figure 5(b)), and GO clustering analysis showed high ES and gene counts for acute-phase response, chemotaxis, and response to cytokine, and the regulation of inflammation factor production had a high ES and large count (Figure 6). However, the Mdown-YNup showed significant enrichment of terms associated with cell division, proliferation, and cellular matrix formation (Figure 5(c)), and GO clustering further identified cell cycle and chromosome segregation. Taken together, Yunnan Baiyao mitigates *S. aureus*-induced skin inflammation by targeting multiple pathways.

### 3.5. Protein-Protein Interaction Network

The background PPI network was established using the control vs. model DEGs, followed by the inclusion of the model vs. YN-treated DEGs and their first neighbors in order to identify the target genes of Yunnan Baiyao.

**Figure 7:**
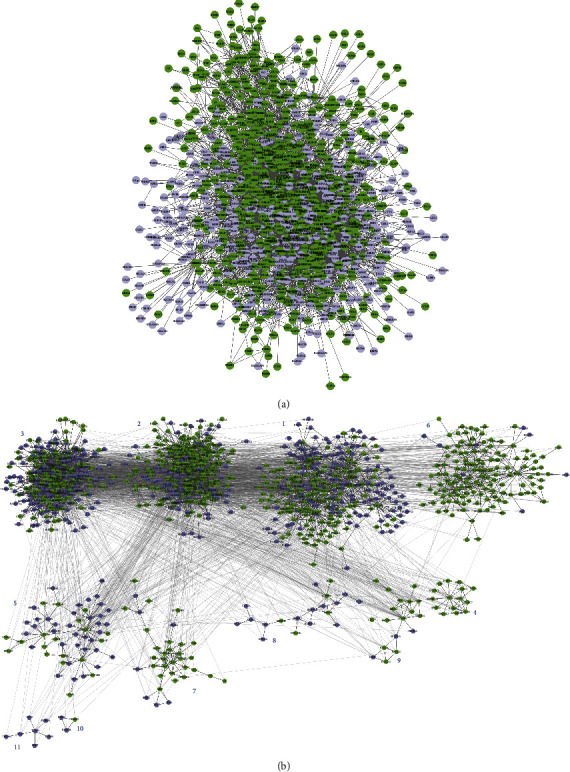
The top modules with relatively high scores selected from the protein-protein interaction network. (a) Model net. (b) Clusters of Model net.

There were 687 nodes (DEGs with a degree higher than 20) in the YN-treated target network that were mapped to the model network (Figure 7(a)). Clustering analysis based on the function of the nodes reveals 11 clusters, of which 4, 6, 9, 7, 2, and 1 were the main mapped targets of the nodes in the YN-treated network in that order (Figure 7(b), and detailed information is presented in Table S4 in supplemental file). The nodes in these clusters were mainly related to cell growth and death (Cluster 4), immune system (Cluster 6), apoptosis (Cluster 9), signal transduction (Cluster 2), and genetic information processing (Cluster 1) based on the KEGG pathway analysis. These network results were consistent with that of GO analysis.

The top 20 nodes of the model and YN-treated networks were screened based on the degree of topology, of which MMP2, PLK1, CCNB1, TLR4, CDK1, CCNA2, CDC25 C, PDGFRA, MYOC, and KNG1 were common to both (Table S5 in Supplemental file) and possibly targeted by Yunnan Baiyao. Furthermore, these ten nodes were related to the cell cycle (5 nodes), oocyte maturation (4 nodes), cell division (4 nodes), protein ubiquitination (3 nodes), and ATP binding (3 nodes). Among the top 20 hub nodes, TLR4 (toll-like receptor 4) and VAV1 (VAV guanine nucleotide exchange factor 1) are the key inflammation genes. TLR4 is a member of the toll-like receptor family, which plays an important role in pathogen recognition and innate immune activation [49]. In many types of animals, TLRs are highly conserved in structure and function across species [49, 50], although they differ in their expression patterns and recognize distinct pathogen-related molecules [51]. TLR4 recognizes LPS of most Gram-negative bacteria and is the key driver of LPS-induced inflammation [52, 53]. VAV1, a member of the VAV gene family, is the guanine nucleotide exchange factor (GEFs) for GTPases of the Rho family, which activates actin cytoskeleton rearrangement [54, 55]. Furthermore, the Vav1-encoded protein is involved in the mechanical peristalsis and migration of neutrophils in inflammatory microvessels and plays a role in the development and activation of T cells and B cells [56]. In addition, CCNA2, CCNB1, PLK1, CDK1, and CDC25 C, which are related to the cell cycle or other proliferation-associated processes, were also altered to some extent by Yunnan Baiyao. Taken together, Yunnan Baiyao can regulate *S. aureus*-induced skin inflammation through immune activation and cell proliferation.

### 3.6. qRT-PCR Confirmation of Differential mRNA Expression

**Figure 8:**
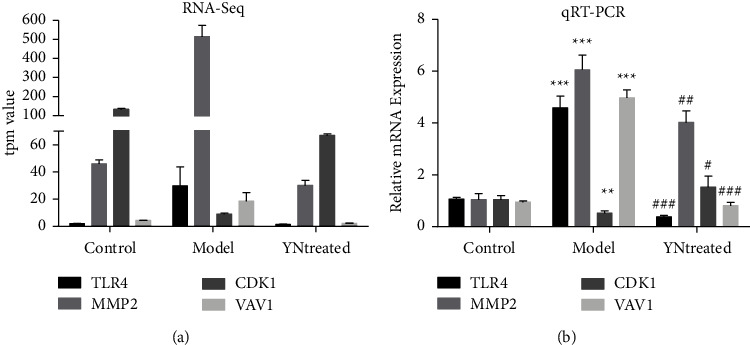
Relative expression of each selected gene validated by qRT-PCR using GAPDH as the housekeeping gene. The expression of each gene relative to GAPDH was calculated using a modified 2^−ΔΔCT^ method. Data represent the mean ± SD of triplicate experiments. ^*∗∗∗*^Control vs model, *P* value < 0.001; ^*∗∗*^control vs model, *P* value < 0.01; ^###^model vs YN-treated, *P* value < 0.001; ^##^model vs YN-treated, *P* value < 0.01; ^#^model vs YN-treated, *P* value < 0.05. (a) TPM values of the selected genes from the RNA-seq data. (b) The relative mRNA expressions of the selected genes from qRT-PCR data.

Expression of MMP2, TLR4, CDK1, and VAV1 was further validated by qRT-PCR and were coincident with RNA-seq data (shown in Figure 8). Thus, both approaches showed a strong correlation.

## 4. Conclusion

In conclusion, we performed a comprehensive bioinformatics analysis on transcriptome data of skin tissue samples obtained from groups of infected animals and Yunnan Baiyao-treated infected animals. The pivotal DEGs and pathways were identified and screened to provide a theoretical basis for the molecular mechanisms involved in skin inflammation and potential drug target discovery. The results showed that Yunnan Baiyao could regulate the inflammatory pathway by changing the expression of various genes so as to alleviate inflammation. Our findings provide insights into the molecular mechanisms underlying bacterial infection-induced skin inflammation and discovery of potential drug targets.

## Figures and Tables

**Table 1:** The specific genes involved in the top 20 enriched GO terms between the model group and the YN-treated group.

No.	Go Terms (Top 20)	Gene counts	*P*-adjust	Gene names
Up-regulated				
1	Mitotic cell cycle process	24	3.30*E* − 07	Birc5; Tpx2; Kif22; Cdkn3; Melk; Kif20a; Ccnb1; Ska3; Cdk1; Plk1; Kif14; Bub1; Iqgap3; Cdc25c; Cdca3; Nuf2; Ccna2; Ccnb2; Cdc20; Ckap2; Ska1; Kifc5b; Nek2; Kif11
2	Cell cycle process	25	8.84*E* − 07	Birc5; Tpx2; Kif22; Cdkn3; Melk; Kif20a; Ccnb1; Ska3; Cdk1; Plk1; Kif14; Bub1; Iqgap3; Ect2; Cdc25c; Cdca3; Nuf2; Ccna2; Ccnb2; Cdc20; Ckap2; Ska1; Kifc5b; Nek2; Kif11
3	Cell division	12	5.27*E* − 05	Ccnb1; Ube2c; Nuf2; Ccna2; Ska3; Ccnb2; Cdc20; Birc5; Cdca2; Ska1; Kifc5b; Cdca3
4	Chromosome segregation	7	3.21*E* − 04	Ska3; Birc5; Cdca2; Plk1; Ska1; Nek2; Kif11
5	Mitotic nuclear division	8	7.57*E* − 04	Ccnb1; Nuf2; Ccna2; Ska3; Ccnb2; Cdc20; Ska1; Cdca3
6	Single-organism cellular process	76	1.13*E* − 03	Tnni3k; Cers4; Ect2; Kif4; Tpx2; Loxhd1; Birc5; Kcne1l; Cdca2; Cdkn3; MVD; St8sia5; Atp4a; Kif22; Gm13502; CHRNG; Melk; Kif20a; Ccnb1; Lmod3; Ska3; Cdk1; Krt9; Krt2; Pyy; Plk1; Lrrc38; Kif14; Alox12e; Bub1; PCLAF; Hist1h2ag; Iqgap3; Plcd4; AC156572.2; Kcna7; Ntrk1; Kcnj16; Kifc5b; Sypl2; Fa2h; Clca1; Slc27a4; Neu2; Aox4; Panx3; Olah; Msmo1; Cdc25c; Akr1c18; Apobec2; CHRNE; Fgf6; Aqp4; Mfsd2a; Cdca3; Gm37035; Slc28a1; Gm3571; Nuf2; Ccna2; Ttc21a; Ccnb2; Cdc20; Idi1; A4gnt; Ckap2; Pon1; Ube2c; Ska1; Elovl3; Spry3; Gm15756; Nek2; Kif11; Cyp17a1
7	Regulation of nuclear division	8	1.19*E* − 03	Ube2c; Birc5; Gm8956; Cdc20; Plk1; Bub1; Nek2; Kif11
8	Lipid biosynthetic process	12	1.56*E* − 03	Fa2h; SQLE; Gm3571; Cers4; Cyp17a1; Olah; Msmo1; MVD; Idi1; Dhcr24; Elovl3; Gm13502
9	Regulation of microtubule polymerization or depolymerization	8	1.56*E* − 03	Ska3; Tpx2; Birc5; Ckap2; Ska1; Kifc5b; Nek2; Kif11
10	Lipid metabolic process	18	1.56*E* − 03	Fa2h; SQLE; Gm3571; Cers4; Pon1; Slc27a4; Plcd4; Cyp17a1; Olah; Msmo1; MVD; Akr1c18; Dhcr24; Elovl3; Neu2; Idi1; Gm13502; Alox12e
11	Microtubule cytoskeleton organization involved in mitosis	5	1.56*E* − 03	Nek2; Tpx2; Birc5; Kif11; Kifc5b
12	Mitotic spindle assembly	5	1.56*E* − 03	Nek2; Tpx2; Birc5; Kif11; Kifc5b
13	Regulation of mitotic nuclear division	7	2.33*E* − 03	Ube2c; Gm8956; Birc5; Plk1; Bub1; Nek2; Kif11
14	Sterol biosynthetic process	5	2.33*E* − 03	Msmo1; MVD; SQLE; Dhcr24; Gm13502
15	Kinesin complex	5	2.33*E* − 03	Kif4; Kif11; Kif20a; Kif22; Kifc5b
16	Microtubule	10	2.33*E* − 03	Kif20a; Ska3; Tpx2; Cdk1; Birc5; Ckap2; Ska1; Kifc5b; Kif11; Kif22
17	Regulation of cell cycle process	12	2.41*E* − 03	Cdk1; Ube2c; Ccna2; Birc5; Tpx2; Gm8956; Cdc20; Plk1; Kif14; Bub1; Nek2; Kif11
18	Cytoskeletal part	20	2.41*E* − 03	Ckap2l; Plk1; Birc5; Kif20a; Ccnb1; Ska3; Tpx2; Cdk1; Cdc20; Kif4; Krt2; Ckap2; Krt9; Ska1; Kifc5b; Nek2; 9430073C21Rik; Kif11; Lmod3; Kif22
19	Polymeric cytoskeletal fiber	12	2.44*E* − 03	Krt2; Kif20a; Ska3; Tpx2; Cdk1; Birc5; Ckap2; Krt9; Ska1; Kifc5b; Kif11; Kif22
20	Supramolecular fiber	12	2.61*E* − 03	Krt2; Kif20a; Ska3; Tpx2; Cdk1; Birc5; Ckap2; Krt9; Ska1; Kifc5b; Kif11; Kif22

Down-regulated				
1	Neutrophil chemotaxis	15	3.22*E* − 08	Csf3r; S100a8; Vav1; JAML; S100a9; Ccl8; Trem3; Ccl6; Ccl9; Il1b; Ccl12; Ccl24; Fcer1g; Ccl11; Ccl7
2	Granulocyte migration	15	3.22*E* − 08	Csf3r; S100a8; Vav1; JAML; S100a9; Ccl11; Trem3; Ccl6; Ccl9; Il1b; Ccl12; Ccl24; Fcer1g; Ccl8; Ccl7
3	Positive regulation of response to wounding	22	3.22*E* − 08	Tlr9; Ptgs2; Ccl9; Fcer1g; Il1rl1; Ccl6; Ccr2; Ccl12; Ccl24; Ccl11; Ccl7; Fcer1a; Tlr11; SELP; Il1b; Ccl8; S100a8; S100a9; Tlr2; Il6; PLEK; Serpine1
4	Chemokine-mediated signaling pathway	18	3.22*E* − 08	Cxcr6; Ccr5; Ccr6; Ccr1; Cxcl1; Cxcl2; Ccl11; PPBP; Ccl6; Ccl9; Ccr2; Ccl12; Ccl24; Cxcr2; Cxcl5; Ccl8; Pf4; Ccl7
5	Activation of immune response	28	3.22*E* − 08	Tlr9; Pik3ap1; C1ra; Lcp2; C1rb; Fcer1g; Btk; Ptpn22; Serping1; Colec12; Themis2; Cd180; Skap1; FCNA; Tmem173; Tlr11; Masp1; Cd79b; Pglyrp1; Nckap1l; C6; Fpr1; Cd14; Tlr2; Havcr2; C5ar1; Klhl6; Tlr4
6	Positive regulation of inflammatory response	20	3.22*E* − 08	Tlr2; S100a9; Fcer1a; Tlr9; Ptgs2; Il1b; Serpine1; S100a8; Tlr11; Ccl11; Il1rl1; Ccl6; Ccl9; Il6; Ccr2; Ccl12; Ccl24; Fcer1g; Ccl8; Ccl7
7	Regulation of inflammatory response	32	3.22*E* − 08	Tnfaip8l2; Tlr9; Ptgs2; Adamts12; Pik3ap1; Ccl9; Fcer1g; Nlrp3; Il12b; Il1rl1; Scgb1a1; Ccl8; Serping1; Ccl6; Ccr2; Ccl12; Ccl24; Ccl11; Ccl7; Il6; Tlr11; Siglec-E; Il1b; Pglyrp1; S100a8; Gm7666; S100a9; Serpine1; Apod; Fcer1a; Tnfaip6; Tlr2
8	Regulation of leukocyte migration	26	3.22*E* − 08	Ccr6; Tnfsf14; Ptpn22; Cxcl13; P2ry12; Ccr2; C3ar1; Ccl7; Ccr1; Gpr18; Cxcl2; SELP; Pla2g7; Rarres2; Cxcl5; Pf4; PPBP; Nckap1l; VEGF-C; Rac2; Serpine1; Cxcl1; GCSAM; Apod; C5ar1; Ccr5
9	Regulation of response to wounding	35	3.22*E* − 08	Il6; Tnfaip8l2; Tlr9; Ptgs2; Ccl11; Adamts12; Pik3ap1; Ccl9; Fcer1g; Nlrp3; Il1rl1; Fcer1a; Ccl8; Serping1; Ccl6; Ccr2; Ccl12; Ccl24; Scara5; Ccl7; Il12b; Tlr11; SELP; Siglec-E; Il1b; Pglyrp1; S100a8; Gm7666; S100a9; Tlr2; Apod; Scgb1a1; Tnfaip6; PLEK; Serpine1
10	Taxis	37	3.22*E* − 08	Bin2; Ccr6; Cyp7b1; Ccl9; Cnr2; PTAFR; Fcer1g; Cxcr6; Coro1a; Sema3a; LBP; Cxcl13; Ccl6; Ccr2; Ccl12; Ccl24; Ccl11; Ccl7; S1pr1; PTPRO; Vav1; PDGFRA; S100a8; Cxcl2; Il1b; Retnlg; Cxcr2; Ccl8; Retnla; Fpr1; JAML; S100a9; Cxcl1; PDGFRB; Trem3; EDNRB; Csf3r
11	Cell chemotaxis	32	3.22*E* − 08	Bin2; Ccr6; Cyp7b1; PTPRO; Ccl9; Cnr2; Fcer1g; Coro1a; PDGFRB; LBP; Ccr2; Ccl12; Ccl24; Ccl11; Ccl7; S1pr1; Ccl6; Vav1; Retnla; Cxcl2; Il1b; Retnlg; Ccl8; Cxcl13; S100a8; Fpr1; JAML; S100a9; Cxcl1; Trem3; EDNRB; Csf3r
12	Positive regulation of defense response	37	3.22*E* − 08	Il6; Tlr9; Cd226; Ptgs2; Pik3ap1; Ccl9; Fcer1g; CYBA; Btk; Pglyrp1; Il1rl1; Ccl6; Colec12; Ccr2; Ccl12; Ccl24; Ccl11; Cd180; Il12b; FCNA; S100a8; Tlr11; Mmp2; Il1b; Ccl8; Tmem173; Tlr4; Cd14; S100a9; Tlr2; Cxcl1; Havcr2; LBP; Vav1; Fcer1a; Ccl7; Serpine1
13	Granulocyte chemotaxis	15	3.22*E* − 08	Csf3r; S100a8; Vav1; JAML; S100a9; Ccl8; Trem3; Ccl6; Ccl9; Il1b; Ccl12; Ccl24; Fcer1g; Ccl11; Ccl7
14	Immune response-regulating signaling pathway	25	3.22*E* − 08	Tlr9; Pik3ap1; Lcp2; Fcer1g; Btk; Ptpn22; Colec12; Themis2; Tlr4; Clec4d; Skap1; FCNA; Tlr11; Ms4a2; Cd79b; Pglyrp1; Nckap1l; Fpr1; Cd14; Tlr2; Havcr2; C5ar1; Fcer1a; Klhl6; Cd180
15	Positive regulation of locomotion	41	3.22*E* − 08	Angpt4; Ccr6; Ptgs2; Cxcl13; Ccr1; Tnfsf14; Coro1a; Sema3a; MYOC; Snai1; Glipr2; Csf1r; Cxcr3; Fermt3; PDPN; Ccr2; C3ar1; Ntn1; Ccl11; Ccl7; SPARC; Cyr61; Srpx2; Cxcl2; Flt4; Pla2g7; Rarres2; SELP; Cxcl5; Col1a1; Pf4; PPBP; Nckap1l; VEGF-C; Rac2; Serpine1; Mmp9; Cxcl1; PDGFRB; C5ar1; Ccr5
16	Positive regulation of cell migration	38	3.22*E* − 08	Angpt4; Ptgs2; Cxcl13; Ccr1; Tnfsf14; Coro1a; Sema3a; MYOC; Snai1; Glipr2; Csf1r; Fermt3; PDPN; Ccr2; C3ar1; Ccl11; Ccl7; SPARC; Cyr61; Srpx2; Cxcl2; Flt4; Pla2g7; Rarres2; SELP; Cxcl5; Col1a1; Pf4; PPBP; Nckap1l; VEGF-C; Rac2; Serpine1; Mmp9; Cxcl1; PDGFRB; C5ar1; Ccr5
17	Locomotion	51	3.22*E* − 08	Ntn1; Ccr6; Cyp7b1; Adamts12; Bin2; Ccl9; Grem1; PDGFRA; Fcer1g; PTAFR; Cnr2; Il12b; Cxcr6; Coro1a; Sema3a; LBP; PDGFRB; Pltp; Loxl2; Ccl6; Ccr2; Ccl12; Ccl24; Ccl11; Ccl7; S1pr1; PTPRO; Vav1; Cyr61; Srpx2; Nckap1l; Cxcl2; S100a8; Cthrc1; Il1b; Retnlg; Cxcr2; Ccl8; Cxcl13; Retnla; Fpr1; JAML; S100a9; Mmp9; Cxcl1; Trem3; Itgb7; EDNRB; Csf3r; Cd84; Lcp1
18	Neutrophil migration	15	3.22*E* − 08	Csf3r; S100a8; Vav1; JAML; S100a9; Ccl8; Trem3; Ccl6; Ccl9; Il1b; Ccl12; Ccl24; Fcer1g; Ccl11; Ccl7
19	Regulation of immune response	57	3.22*E* − 08	Btk; Cd226; HCST; Sash3; Havcr2; Pik3ap1; C1ra; LPXN; Lcp2; Fcer1g; C1rb; Skap1; Tlr9; Ptpn22; Serping1; FCRLB; Pglyrp1; Il1rl1; LBP; Colec12; Ccr2; Il27ra; CYBA; Tnfsf13b; Masp1; Cd180; Clec4d; Cd14; Vav1; Ccr1; FCNA; Themis2; Nckap1l; Tlr11; Mmp2; Ms4a2; Cd79b; Il6; Slamf1; Il12b; Tmem173; Gm7666; Tlr4; Fpr1; C6; Gfi1; Rac2; Il7r; Tlr2; GCSAM; Nlrp3; C5ar1; Slc11a1; Fcer1a; Klhl6; Cmtm3; Cd84
20	Cytokine-mediated signaling pathway	31	3.22*E* − 08	Ccr6; Il10ra; Ccl9; Ebi3; Grem2; Csf1r; Il2rb; Il1rl1; Ccl6; Pf4; Ccr2; Ccl12; Ccl24; Aspn; Ccl11; Ccl7; Cxcr6; Il12b; Ccr1; Cxcl2; Il1b; Cxcr2; Cxcl5; Ccl8; Rtn4rl2; PPBP; Il7r; Cxcl1; Il6; Csf3r; Ccr5

## Data Availability

All raw sequencing reads have been submitted to the NCBI Sequence Read Archive (https://www.ncbi.nlm.nih.gov/sra) under the BioProject accession number PRJNA836085.

## Supplementary Materials

Supplementary Materials The Supplementary Material for this article included three supplementary figures (Figure S1, Figure I, and Figure II), eight supplementary tables (Table S1–S6, Table I, and Table II), and a brief result of the analysis of DEGs in the inflamed skin samples relative to the normal skin as listed in the following paragraph, which were published before. Plus, Figure I, Figure II, Table I, and Table II were belonging to the brief analysis published before. Besides, a graphic bbstract was added in the Supplementary Material too. Figure S1. The results of the KEGG pathway data of upregulated (A) and downregulated (B) YN-treated DEGs. Figure I. The top 20 GO terms (A) and KEGG pathways (B) of different expressed genes enrichment in RNA-sequencing (*P* value ≤ 0.05). Gene counts: the number of target genes in each GO term or the KEGG pathway. Rich factor: the ratio of the number of target genes divided by the number of all the genes in each GO term or the KEGG pathway. The size of pot indicated the gene counts, and the color reflected the different *P* value. Figure II. GO clustering results of DEGs with a criterion of the enrichment score >1. A, upregulated. B, downregulated. Counts represent the gene number enriched in each term. The enrichment score was obtained according to the built-in program in DAVID, which ranks the biological significance of gene groups based on the overall *P* value of all enriched annotation terms. The higher the enrichment score is, the more important the term is. Table S1. Summary of trimming and read mapping results of the sequences generated from the skin samples. Table S2. 201 genes upregulated in the model while downregulated by Yunnan Baiyao (known as Mup-YNdown). Table S3. 76 genes downregulated in the model while upregulated by Yunnan Baiyao (known as Mdown-YNup). Table S4. Detailed information of YN-treated nodes mapped in each cluster. Table S5. The top 20 nodes in the Model net and YN-treated net. Table S6. Primer sequences for real-time PCR. Table I. Main GO and pathway enrichment analysis relating to all hubs. Table II. Pathway enrichment analysis of PPI clusters related to major hubs (Top 5).
