# Supplementary material for: RNA Sequencing Reveals the Regulation Mechanism of Yunnan Baiyao in Treating Skin Infection Caused by Staphylococcus aureus
Source: Evid Based Complement Alternat Med. 2022 Oct 12;2022:6348157. doi: 10.1155/2022/6348157 (PMC9581712; doi:10.1155/2022/6348157)
Supplement: Supplementary Materials — The Supplementary Material for this article included three supplementary figures (Figure S1, Figure I, and Figure II), eight supplementary tables (Table S1–S6, Table I, and Table II), and a brief result of the analysis of DEGs in the inflamed skin samples relative to the normal skin as listed in the following paragraph, which were published before. Plus, Figure I, Figure II, Table I, and Table II were belonging to the brief analysis published before. Besides, a graphic bbstract was added in the Supplementary Material too. Figure S1. The results of the KEGG pathway data of upregulated (A) and downregulated (B) YN-treated DEGs. Figure I. The top 20 GO terms (A) and KEGG pathways (B) of different expressed genes enrichment in RNA-sequencing (P value ≤ 0.05). Gene counts: the number of target genes in each GO term or the KEGG pathway. Rich factor: the ratio of the number of target genes divided by the number of all the genes in each GO term or the KEGG pathway. The size of pot indicated the gene counts, and the color reflected the different P value. Figure II. GO clustering results of DEGs with a criterion of the enrichment score >1. A, upregulated. B, downregulated. Counts represent the gene number enriched in each term. The enrichment score was obtained according to the built-in program in DAVID, which ranks the biological significance of gene groups based on the overall P value of all enriched annotation terms. The higher the enrichment score is, the more important the term is. Table S1. Summary of trimming and read mapping results of the sequences generated from the skin samples. Table S2. 201 genes upregulated in the model while downregulated by Yunnan Baiyao (known as Mup-YNdown). Table S3. 76 genes downregulated in the model while upregulated by Yunnan Baiyao (known as Mdown-YNup). Table S4. Detailed information of YN-treated nodes mapped in each cluster. Table S5. The top 20 nodes in the Model net and YN-treated net. Table S6. Primer sequences for real-time PCR. [file 6348157.f1.docx]

**Graphical Abstract**

Yunnan Baiyao can significantly relieve inflammatory symptoms induced by Staphylococcus aureus, by regulating genes and pathways involved in the following processes such as accelerating cell cycle process and metabolism, suppressing inflammation factors production and acute-phase response.


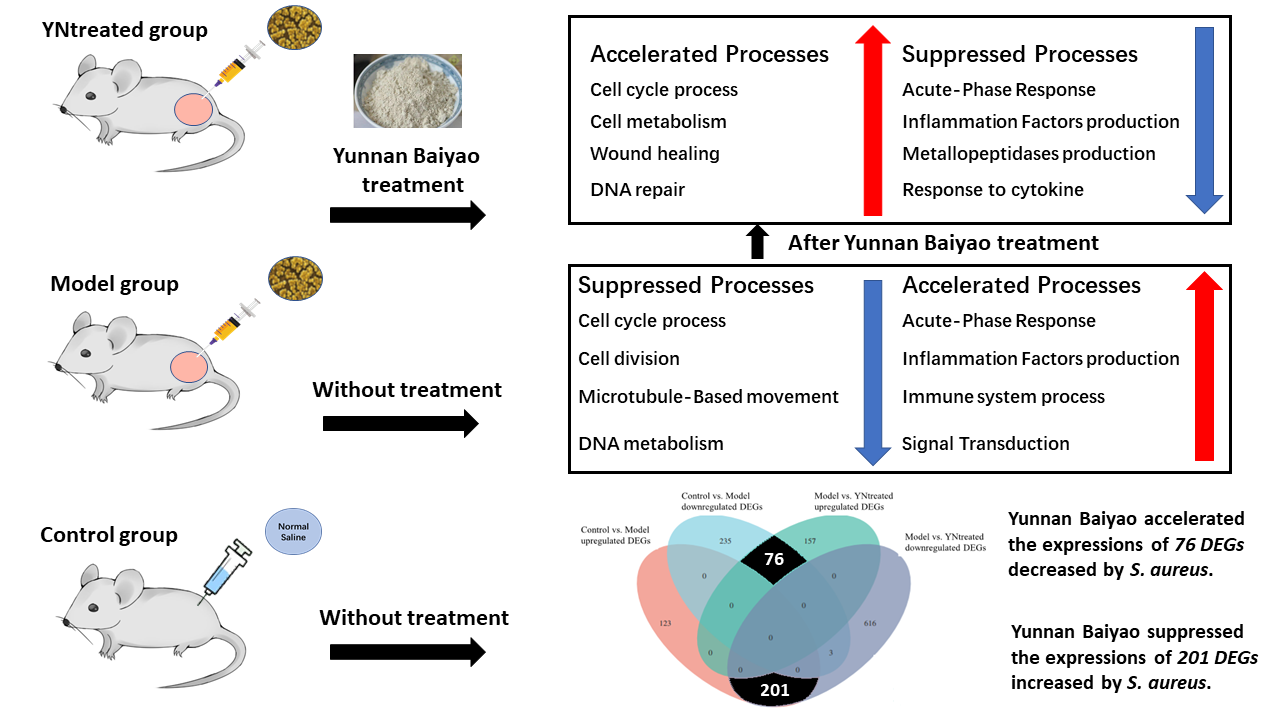


**Figure S1. The results of the KEGG pathway data of upregulated (A) and downregulated(B) YNtreated DEGs.**

**A**

**
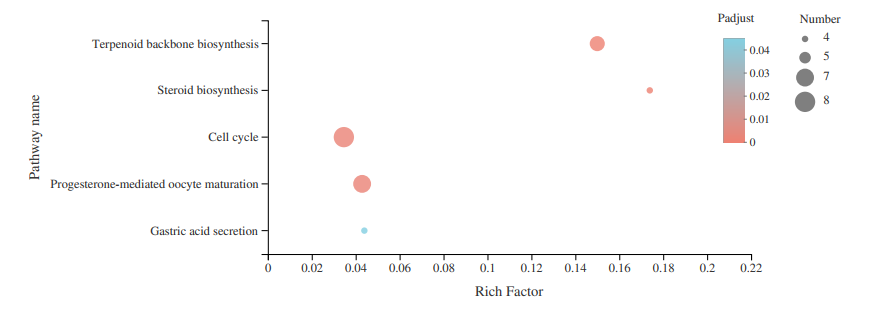
**

**B**

**
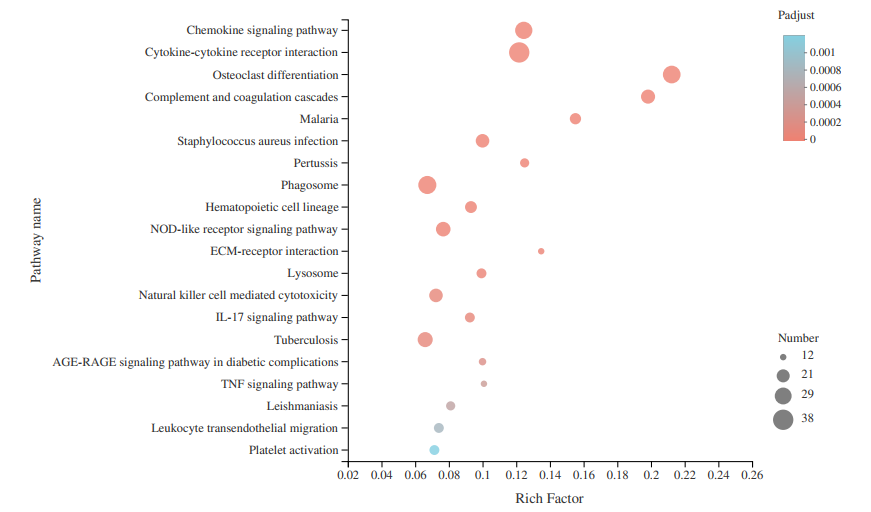
**

**Table S1. Summary of trimming and read mapping results of the sequences generated from the skin samples.**

| Sample | Raw reads | Clean reads | Total reads | Total mapped | Multiple mapped | Uniquely mapped |
| --- | --- | --- | --- | --- | --- | --- |
| Control1 | 45,022,032 | 44,445,246 | 44,445,246 | 42,720,677(96.12%) | 3,242,878(7.3%) | 39,477,799(88.82%) |
| Control2 | 44,633,900 | 44,348,374 | 44,348,374 | 42,988,527(96.93%) | 3,280,302(7.4%) | 39,708,225(89.54%) |
| Control3 | 44,768,108 | 44,227,922 | 44,227,922 | 42,503,298(96.1%) | 3,238,662(7.32%) | 39,264,636(88.78%) |
| Model1 | 43,619,658 | 43,337,052 | 43,337,052 | 41,713,031(96.25%) | 2,676,494(6.18%) | 39,036,537(90.08%) |
| Model2 | 44,876,888 | 44,364,064 | 44,364,064 | 42,687,638(96.22%) | 2,632,583(5.93%) | 40,055,055(90.29%) |
| Model3 | 44,517,200 | 44,270,602 | 44,270,602 | 42,717,763(96.49%) | 2,922,110(6.6%) | 39,795,653(89.89%) |
| YNtreated1 | 44,532,750 | 44,062,456 | 44,062,456 | 42,267,084(95.93%) | 3,137,213(7.12%) | 39,129,871(88.81%) |
| YNtreated2 | 44,458,372 | 43,974,492 | 43,974,492 | 42,163,358(95.88%) | 3,196,182(7.27%) | 38,967,176(88.61%) |
| YNtreated3 | 47,105,610 | 46,766,168 | 46,766,168 | 44,879,562(95.97%) | 3,343,829(7.15%) | 41,535,733(88.82%) |

**Note**: Control represents the skin sample of mice without the injection of *S. aureus*; Model represents the skin sample of mice with the injection of *S. aureus*. YNtreated represents the skin sample of mice with Yunnan Baiyao treatment after the injection of *S. aureus*. Three replicates of Control (Control-1, -2 and -3), Model (Model-1, -2 and -3) and YNtreated (YNtreated-1, -2 and -3) treatments were carried out in RNA-seq analysis.

**Table S2. 201 Genes upregulated in the Model while downregulated by Yunnan Baiyao (known as Mup-YNdown)**

| ID | Gene Name | Function |
| --- | --- | --- |
| ENSMUSG00000030787 | lymphatic vessel endothelial hyaluronan receptor 1(Lyve1) | Transportation |
| ENSMUSG00000027219 | solute carrier family 28 (sodium-coupled nucleoside transporter), member 2(Slc28a2) | Transportation |
| ENSMUSG00000022032 | scavenger receptor class A, member 5(Scara5) | Transportation |
| ENSMUSG00000059654 | regenerating islet-derived 1(Reg1) | Transportation |
| ENSMUSG00000045404 | potassium channel, subfamily K, member 13(Kcnk13) | Transportation |
| ENSMUSG00000024730 | membrane-spanning 4-domains, subfamily A, member 8A(Ms4a8a) | Transportation |
| ENSMUSG00000024672 | membrane-spanning 4-domains, subfamily A, member 7(Ms4a7) | Transportation |
| ENSMUSG00000024679 | membrane-spanning 4-domains, subfamily A, member 6D(Ms4a6d) | Transportation |
| ENSMUSG00000079419 | membrane-spanning 4-domains, subfamily A, member 6C(Ms4a6c) | Transportation |
| ENSMUSG00000024677 | membrane-spanning 4-domains, subfamily A, member 6B(Ms4a6b) | Transportation |
| ENSMUSG00000024680 | membrane-spanning 4-domains, subfamily A, member 2(Ms4a2) | Transportation |
| ENSMUSG00000074109 | MAS-related GPR, member X2(Mrgprx2) | Transportation |
| ENSMUSG00000050276 | MAS-related GPR, member G(Mrgprg) | Transportation |
| ENSMUSG00000070547 | MAS-related GPR, member B1(Mrgprb1) | Transportation |
| ENSMUSG00000020357 | FMS-like tyrosine kinase 4(Flt4) | Transportation |
| ENSMUSG00000027656 | cellular communication network factor 5(Ccn5) | Transportation |
| ENSMUSG00000020709 | ArfGAP with dual PH domains 2(Adap2) | Transportation |
| ENSMUSG00000029778 | adenylate cyclase activating polypeptide 1 receptor 1(Adcyap1r1) | Transportation |
| ENSMUSG00000026725 | tenascin N(Tnn) | Structure dermal components |
| ENSMUSG00000029231 | platelet derived growth factor receptor, alpha polypeptide(Pdgfra) | Structure dermal components |
| ENSMUSG00000027832 | pentraxin related gene(Ptx3) | Structure dermal components |
| ENSMUSG00000048368 | osteomodulin(Omd) | Structure dermal components |
| ENSMUSG00000026697 | myocilin(Myoc) | Structure dermal components |
| ENSMUSG00000021596 | multiple C2 domains, transmembrane 1(Mctp1) | Structure dermal components |
| ENSMUSG00000005800 | matrix metallopeptidase 8(Mmp8) | Structure dermal components |
| ENSMUSG00000031740 | matrix metallopeptidase 2(Mmp2) | Structure dermal components |
| ENSMUSG00000000901 | matrix metallopeptidase 11(Mmp11) | Structure dermal components |
| ENSMUSG00000024481 | laeverin(Lvrn) | Structure dermal components |
| ENSMUSG00000076609 | immunoglobulin kappa constant(Igkc) | Structure dermal components |
| ENSMUSG00000026938 | ficolin A(Fcna) | Structure dermal components |
| ENSMUSG00000025551 | fibroblast growth factor 14(Fgf14) | Structure dermal components |
| ENSMUSG00000019278 | dipeptidase 1(Dpep1) | Structure dermal components |
| ENSMUSG00000073418 | complement component 4B (Chido blood group)(C4b) | Structure dermal components |
| ENSMUSG00000036896 | complement component 1, q subcomponent, C chain(C1qc) | Structure dermal components |
| ENSMUSG00000036905 | complement component 1, q subcomponent, beta polypeptide(C1qb) | Structure dermal components |
| ENSMUSG00000036887 | complement component 1, q subcomponent, alpha polypeptide(C1qa) | Structure dermal components |
| ENSMUSG00000046318 | collagen and calcium binding EGF domains 1(Ccbe1) | Structure dermal components |
| ENSMUSG00000028111 | cathepsin K(Ctsk) | Structure dermal components |
| ENSMUSG00000042254 | cartilage intermediate layer protein, nucleotide pyrophosphohydrolase(Cilp) | Structure dermal components |
| ENSMUSG00000001865 | carboxypeptidase A3, mast cell(Cpa3) | Structure dermal components |
| ENSMUSG00000058914 | C1q and tumor necrosis factor related protein 3(C1qtnf3) | Structure dermal components |
| ENSMUSG00000020681 | angiotensin I converting enzyme (peptidyl-dipeptidase A) 1(Ace) | Structure dermal components |
| ENSMUSG00000033453 | a disintegrin-like and metallopeptidase (reprolysin type) with thrombospondin type 1 motif, 15(Adamts15) | Structure dermal components |
| ENSMUSG00000040046 | tryptophan hydroxylase 1(Tph1) | Oxidation-reduction process |
| ENSMUSG00000028011 | tryptophan 2,3-dioxygenase(Tdo2) | Oxidation-reduction process |
| ENSMUSG00000029925 | thromboxane A synthase 1, platelet(Tbxas1) | Oxidation-reduction process |
| ENSMUSG00000030562 | NADPH oxidase 4(Nox4) | Oxidation-reduction process |
| ENSMUSG00000089694 | N-acetyltransferase 8 (GCN5-related) family member 7(Nat8f7) | Oxidation-reduction process |
| ENSMUSG00000051262 | N-acetyltransferase 8 (GCN5-related) family member 3(Nat8f3) | Oxidation-reduction process |
| ENSMUSG00000024529 | lysyl oxidase(Lox) | Oxidation-reduction process |
| ENSMUSG00000024827 | glycine decarboxylase(Gldc) | Oxidation-reduction process |
| ENSMUSG00000018339 | glutathione peroxidase 3(Gpx3) | Oxidation-reduction process |
| ENSMUSG00000040170 | flavin containing monooxygenase 2(Fmo2) | Oxidation-reduction process |
| ENSMUSG00000003617 | ceruloplasmin(Cp) | Oxidation-reduction process |
| ENSMUSG00000028167 | 3-hydroxybutyrate dehydrogenase, type 2(Bdh2) | Oxidation-reduction process |
| ENSMUSG00000034645 | zyg-11 family member A, cell cycle regulator(Zyg11a) | Metabolism/Cell proliferation/Regulation |
| ENSMUSG00000033825 | tryptase beta 2(Tpsb2) | Metabolism/Cell proliferation/Regulation |
| ENSMUSG00000024173 | tryptase alpha/beta 1(Tpsab1) | Metabolism/Cell proliferation/Regulation |
| ENSMUSG00000028834 | tripartite motif-containing 63(Trim63) | Metabolism/Cell proliferation/Regulation |
| ENSMUSG00000052749 | tripartite motif-containing 30B(Trim30b) | Metabolism/Cell proliferation/Regulation |
| ENSMUSG00000048572 | transmembrane protein 252(Tmem252) | Metabolism/Cell proliferation/Regulation |
| ENSMUSG00000069763 | transmembrane protein 100(Tmem100) | Metabolism/Cell proliferation/Regulation |
| ENSMUSG00000039037 | ST6 (alpha-N-acetyl-neuraminyl-2,3-beta-galactosyl-1,3)-N-acetylgalactosaminide alpha-2,6-sialyltransferase 5(St6galnac5) | Metabolism/Cell proliferation/Regulation |
| ENSMUSG00000022372 | src-like adaptor(Sla) | Metabolism/Cell proliferation/Regulation |
| ENSMUSG00000030474 | sialic acid binding Ig-like lectin E(Siglece) | Metabolism/Cell proliferation/Regulation |
| ENSMUSG00000027322 | sialic acid binding Ig-like lectin 1, sialoadhesin(Siglec1) | Metabolism/Cell proliferation/Regulation |
| ENSMUSG00000064373 | selenoprotein P(Selenop) | Metabolism/Cell proliferation/Regulation |
| ENSMUSG00000026580 | selectin, platelet(Selp) | Metabolism/Cell proliferation/Regulation |
| ENSMUSG00000026581 | selectin, lymphocyte(Sell) | Metabolism/Cell proliferation/Regulation |
| ENSMUSG00000022651 | resistin like gamma(Retnlg) | Metabolism/Cell proliferation/Regulation |
| ENSMUSG00000061100 | resistin like alpha(Retnla) | Metabolism/Cell proliferation/Regulation |
| ENSMUSG00000055069 | RAB39, member RAS oncogene family(Rab39) | Metabolism/Cell proliferation/Regulation |
| ENSMUSG00000039126 | prune homolog 2(Prune2) | Metabolism/Cell proliferation/Regulation |
| ENSMUSG00000006014 | proteoglycan 4 (megakaryocyte stimulating factor, articular superficial zone protein)(Prg4) | Metabolism/Cell proliferation/Regulation |
| ENSMUSG00000024180 | post-glycosylphosphatidylinositol attachment to proteins 6(Pgap6) | Metabolism/Cell proliferation/Regulation |
| ENSMUSG00000021903 | polypeptide N-acetylgalactosaminyltransferase 15(Galnt15) | Metabolism/Cell proliferation/Regulation |
| ENSMUSG00000029322 | placenta-specific 8(Plac8) | Metabolism/Cell proliferation/Regulation |
| ENSMUSG00000027188 | peptidase domain containing associated with muscle regeneration 1(Pamr1) | Metabolism/Cell proliferation/Regulation |
| ENSMUSG00000039057 | myosin XVI(Myo16) | Metabolism/Cell proliferation/Regulation |
| ENSMUSG00000042485 | musculoskeletal, embryonic nuclear protein 1(Mustn1) | Metabolism/Cell proliferation/Regulation |
| ENSMUSG00000054641 | multimerin 1(Mmrn1) | Metabolism/Cell proliferation/Regulation |
| ENSMUSG00000105557 | microRNA 3966(Mir3966) | Metabolism/Cell proliferation/Regulation |
| ENSMUSG00000024678 | membrane-spanning 4-domains, subfamily A, member 4D(Ms4a4d) | Metabolism/Cell proliferation/Regulation |
| ENSMUSG00000026712 | mannose receptor, C type 1(Mrc1) | Metabolism/Cell proliferation/Regulation |
| ENSMUSG00000039252 | leucine-rich repeat LGI family, member 2(Lgi2) | Metabolism/Cell proliferation/Regulation |
| ENSMUSG00000023046 | insulin-like growth factor binding protein 6(Igfbp6) | Metabolism/Cell proliferation/Regulation |
| ENSMUSG00000003477 | indolethylamine N-methyltransferase(Inmt) | Metabolism/Cell proliferation/Regulation |
| ENSMUSG00000004328 | hypoxia inducible factor 3, alpha subunit(Hif3a) | Metabolism/Cell proliferation/Regulation |
| ENSMUSG00000020251 | glycosyltransferase 8 domain containing 2(Glt8d2) | Metabolism/Cell proliferation/Regulation |
| ENSMUSG00000031162 | GATA binding protein 1(Gata1) | Metabolism/Cell proliferation/Regulation |
| ENSMUSG00000000182 | fibroblast growth factor 23(Fgf23) | Metabolism/Cell proliferation/Regulation |
| ENSMUSG00000047992 | divergent protein kinase domain 1C(Dipk1c) | Metabolism/Cell proliferation/Regulation |
| ENSMUSG00000030147 | C-type lectin domain family 4, member b1(Clec4b1) | Metabolism/Cell proliferation/Regulation |
| ENSMUSG00000043832 | C-type lectin domain family 4, member a3(Clec4a3) | Metabolism/Cell proliferation/Regulation |
| ENSMUSG00000000318 | C-type lectin domain family 10, member A(Clec10a) | Metabolism/Cell proliferation/Regulation |
| ENSMUSG00000057191 | cDNA sequence AB124611(AB124611) | Metabolism/Cell proliferation/Regulation |
| ENSMUSG00000029082 | bone marrow stromal cell antigen 1(Bst1) | Metabolism/Cell proliferation/Regulation |
| ENSMUSG00000021388 | asporin(Aspn) | Metabolism/Cell proliferation/Regulation |
| ENSMUSG00000002992 | apolipoprotein C2(Apoc2) | Metabolism/Cell proliferation/Regulation |
| ENSMUSG00000042607 | ankyrin repeat and SOCS box-containing 4(Asb4) | Metabolism/Cell proliferation/Regulation |
| ENSMUSG00000033544 | angiopoietin-like 1(Angptl1) | Metabolism/Cell proliferation/Regulation |
| ENSMUSG00000054204 | ALK and LTK ligand 2(Alkal2) | Metabolism/Cell proliferation/Regulation |
| ENSMUSG00000030669 | calcitonin/calcitonin-related polypeptide, alpha(Calca) | Metabolism/Cell proliferation/Regulation |
| ENSMUSG00000050345 | 4930486L24Rik | Metabolism/Cell proliferation/Regulation |
| ENSMUSG00000015354 | procollagen C-endopeptidase enhancer 2(Pcolce2) | Lipid metabolism |
| ENSMUSG00000041797 | ATP-binding cassette, sub-family A (ABC1), member 9(Abca9) | Lipid metabolism |
| ENSMUSG00000044749 | ATP-binding cassette, sub-family A (ABC1), member 6(Abca6) | Lipid metabolism |
| ENSMUSG00000032081 | apolipoprotein C-III(Apoc3) | Lipid metabolism |
| ENSMUSG00000069792 | WAP four-disulfide core domain 17(Wfdc17) | Immune function |
| ENSMUSG00000044206 | V-set and immunoglobulin domain containing 4(Vsig4) | Immune function |
| ENSMUSG00000071068 | triggering receptor expressed on myeloid cells-like 2(Treml2) | Immune function |
| ENSMUSG00000042265 | triggering receptor expressed on myeloid cells 1(Trem1) | Immune function |
| ENSMUSG00000040522 | toll-like receptor 8(Tlr8) | Immune function |
| ENSMUSG00000044583 | toll-like receptor 7(Tlr7) | Immune function |
| ENSMUSG00000039005 | toll-like receptor 4(Tlr4) | Immune function |
| ENSMUSG00000055546 | T cell immunoglobulin and mucin domain containing 4(Timd4) | Immune function |
| ENSMUSG00000040026 | serum amyloid A 3(Saa3) | Immune function |
| ENSMUSG00000057465 | serum amyloid A 2(Saa2) | Immune function |
| ENSMUSG00000074115 | serum amyloid A 1(Saa1) | Immune function |
| ENSMUSG00000021091 | serine (or cysteine) peptidase inhibitor, clade A, member 3N(Serpina3n) | Immune function |
| ENSMUSG00000079012 | serine (or cysteine) peptidase inhibitor, clade A, member 3M(Serpina3m) | Immune function |
| ENSMUSG00000058207 | serine (or cysteine) peptidase inhibitor, clade A, member 3K(Serpina3k) | Immune function |
| ENSMUSG00000028883 | sema domain, immunoglobulin domain (Ig), short basic domain, secreted, (semaphorin) 3A(Sema3a) | Immune function |
| ENSMUSG00000024653 | secretoglobin, family 1A, member 1 (uteroglobin)(Scgb1a1) | Immune function |
| ENSMUSG00000056071 | S100 calcium binding protein A9 (calgranulin B)(S100a9) | Immune function |
| ENSMUSG00000056054 | S100 calcium binding protein A8 (calgranulin A)(S100a8) | Immune function |
| ENSMUSG00000069893 | RIKEN cDNA 9930111J21 gene 1(9930111J21Rik1) | Immune function |
| ENSMUSG00000019577 | pyruvate dehydrogenase kinase, isoenzyme 4(Pdk4) | Immune function |
| ENSMUSG00000008496 | POU domain, class 2, transcription factor 2(Pou2f2) | Immune function |
| ENSMUSG00000025017 | phosphoinositide-3-kinase adaptor protein 1(Pik3ap1) | Immune function |
| ENSMUSG00000058818 | paired Ig-like receptor B(Pirb) | Immune function |
| ENSMUSG00000026442 | neurofascin(Nfasc) | Immune function |
| ENSMUSG00000022887 | mannan-binding lectin serine peptidase 1(Masp1) | Immune function |
| ENSMUSG00000004707 | lymphocyte antigen 9(Ly9) | Immune function |
| ENSMUSG00000071656 | LRRN4 C-terminal like(Lrrn4cl) | Immune function |
| ENSMUSG00000016024 | lipopolysaccharide binding protein(Lbp) | Immune function |
| ENSMUSG00000026822 | lipocalin 2(Lcn2) | Immune function |
| ENSMUSG00000037095 | leucine-rich alpha-2-glycoprotein 1(Lrg1) | Immune function |
| ENSMUSG00000060459 | kininogen 2(Kng2) | Immune function |
| ENSMUSG00000043008 | kelch-like 6(Klhl6) | Immune function |
| ENSMUSG00000048534 | junction adhesion molecule like(Jaml) | Immune function |
| ENSMUSG00000026069 | interleukin 1 receptor-like 1(Il1rl1) | Immune function |
| ENSMUSG00000059108 | interferon induced transmembrane protein 6(Ifitm6) | Immune function |
| ENSMUSG00000073490 | interferon activated gene 207(Ifi207) | Immune function |
| ENSMUSG00000054203 | interferon activated gene 205(Ifi205) | Immune function |
| ENSMUSG00000030786 | integrin alpha M(Itgam) | Immune function |
| ENSMUSG00000031722 | haptoglobin(Hp) | Immune function |
| ENSMUSG00000074934 | gremlin 1, DAN family BMP antagonist(Grem1) | Immune function |
| ENSMUSG00000046856 | G protein-coupled receptor 1(Gpr1) | Immune function |
| ENSMUSG00000059089 | Fc receptor, IgG, low affinity IV(Fcgr4) | Immune function |
| ENSMUSG00000005339 | Fc receptor, IgE, high affinity I, alpha polypeptide(Fcer1a) | Immune function |
| ENSMUSG00000074874 | cytotoxic T lymphocyte-associated protein 2 beta(Ctla2b) | Immune function |
| ENSMUSG00000044258 | cytotoxic T lymphocyte-associated protein 2 alpha(Ctla2a) | Immune function |
| ENSMUSG00000026365 | complement component factor h(Cfh) | Immune function |
| ENSMUSG00000079105 | complement component 7(C7) | Immune function |
| ENSMUSG00000022181 | complement component 6(C6) | Immune function |
| ENSMUSG00000049130 | complement component 5a receptor 1(C5ar1) | Immune function |
| ENSMUSG00000039109 | coagulation factor XIII, A1 subunit(F13a1) | Immune function |
| ENSMUSG00000026180 | chemokine (C-X-C motif) receptor 2(Cxcr2) | Immune function |
| ENSMUSG00000029371 | chemokine (C-X-C motif) ligand 5(Cxcl5) | Immune function |
| ENSMUSG00000023078 | chemokine (C-X-C motif) ligand 13(Cxcl13) | Immune function |
| ENSMUSG00000019122 | chemokine (C-C motif) ligand 9(Ccl9) | Immune function |
| ENSMUSG00000018927 | chemokine (C-C motif) ligand 6(Ccl6) | Immune function |
| ENSMUSG00000015854 | CD5 antigen-like(Cd5l) | Immune function |
| ENSMUSG00000048498 | CD300E molecule(Cd300e) | Immune function |
| ENSMUSG00000034652 | CD300A molecule(Cd300a) | Immune function |
| ENSMUSG00000069609 | CD300 molecule like family member D4(Cd300ld4) | Immune function |
| ENSMUSG00000069607 | CD300 molecule like family member D3(Cd300ld3) | Immune function |
| ENSMUSG00000034028 | CD226 antigen(Cd226) | Immune function |
| ENSMUSG00000079168 | CD209g antigen(Cd209g) | Immune function |
| ENSMUSG00000040197 | CD209e antigen(Cd209e) | Immune function |
| ENSMUSG00000031495 | CD209d antigen(Cd209d) | Immune function |
| ENSMUSG00000052212 | CD177 antigen(Cd177) | Immune function |
| ENSMUSG00000008845 | CD163 antigen(Cd163) | Immune function |
| ENSMUSG00000024803 | ankyrin repeat domain 1 (cardiac muscle)(Ankrd1) | Immune function |
| ENSMUSG00000030790 | adrenomedullin(Adm) | Immune function |
| ENSMUSG00000004730 | adhesion G protein-coupled receptor E1(Adgre1) | Immune function |
| ENSMUSG00000066861 | 2'-5' oligoadenylate synthetase 1G(Oas1g) | Immune function |
| ENSMUSG00000053765 | 2'-5' oligoadenylate synthetase 1F(Oas1f) | Immune function |
| ENSMUSG00000052776 | 2'-5' oligoadenylate synthetase 1A(Oas1a) | Immune function |
| ENSMUSG00000028369 | sushi, von Willebrand factor type A, EGF and pentraxin domain containing 1(Svep1) | Cell adhesion |
| ENSMUSG00000051242 | protocadherin beta 9(Pcdhb9) | Cell adhesion |
| ENSMUSG00000063011 | Mesothelin (Msln) | Cell adhesion |
| ENSMUSG00000032925 | integrin, beta-like 1(Itgbl1) | Cell adhesion |
| ENSMUSG00000005338 | cell adhesion molecule 3(Cadm3) | Cell adhesion |
| ENSMUSG00000040078 | Gm9769 | Function unknown |
| ENSMUSG00000067547 | Gm7666 | Function unknown |
| ENSMUSG00000079559 | Gm684 | Function unknown |
| ENSMUSG00000053541 | Gm4759 | Function unknown |
| ENSMUSG00000105245 | Gm43076 | Function unknown |
| ENSMUSG00000074417 | Gm14548 | Function unknown |
| ENSMUSG00000071036 | Gm10309 | Function unknown |
| ENSMUSG00000078122 | F630028O10Rik | Function unknown |
| ENSMUSG00000099757 | expressed sequence BE692007(BE692007) | Function unknown |
| ENSMUSG00000043740 | B430306N03Rik | Function unknown |
| ENSMUSG00000038768 | 9130409I23Rik | Function unknown |
| ENSMUSG00000106157 | 4930555A03Rik | Function unknown |
| ENSMUSG00000044694 | 2010007H06Rik | Function unknown |
| ENSMUSG00000074677 | signal-regulatory protein beta 1C(Sirpb1c) | Function unknown |
| ENSMUSG00000047420 | family with sequence similarity 180, member A(Fam180a) | Function unknown |
| ENSMUSG00000035184 | family with sequence similarity 124, member A(Fam124a) | Function unknown |

**Table S3. 76 Genes downregulated in the Model while upregulated by Yunnan Baiyao (known as Mdown-YNup)**

| ID | Gene Name | Function |
| --- | --- | --- |
| ENSMUSG00000020330 | hyaluronan mediated motility receptor (RHAMM)(Hmmr) | Transportation |
| ENSMUSG00000024411 | aquaporin 4(Aqp4) | Transportation |
| ENSMUSG00000094793 | major urinary protein 12(Mup12) | Transportation |
| ENSMUSG00000090122 | potassium voltage-gated channel, Isk-related family, member 1-like, pseudogene (Kcne1l) | Transportation |
| ENSMUSG00000085837 | potassium large conductance calcium-activated channel, subfamily M, beta member 4, opposite strand 2（Kcnmb4os2） | Transportation |
| ENSMUSG00000078673 | major urinary protein 19(Mup19) | Transportation |
| ENSMUSG00000069581 | thrombospondin type laminin G domain and EAR repeats(Tspear) | Transportation |
| ENSMUSG00000041498 | kinesin family member 14(Kif14) | Transportation |
| ENSMUSG00000033952 | asp (abnormal spindle)-like, microcephaly associated (Drosophila)(Aspm) | Transportation |
| ENSMUSG00000028655 | major facilitator superfamily domain containing 2A(Mfsd2a) | Transportation |
| ENSMUSG00000027469 | TPX2, microtubule-associated(Tpx2) | Transportation |
| ENSMUSG00000026622 | NIMA (never in mitosis gene a)-related expressed kinase 2(Nek2) | Transportation |
| ENSMUSG00000024873 | cornichon family AMPA receptor auxiliary protein 2(Cnih2) | Transportation |
| ENSMUSG00000024301 | kinesin family member C5B(Kifc5b) | Transportation |
| ENSMUSG00000011118 | pannexin 3(Panx3) | Transportation |
| ENSMUSG00000003779 | kinesin family member 20A(Kif20a) | Transportation |
| ENSMUSG00000012443 | kinesin family member 11(Kif11) | Transportation |
| ENSMUSG00000034311 | kinesin family member 4(Kif4) | Transportation |
| ENSMUSG00000030677 | kinesin family member 22(Kif22) | Transportation |
| ENSMUSG00000051378 | kinesin family member 18B(Kif18b) | Transportation |
| ENSMUSG00000064201 | keratin 2(Krt2) | Structure dermal components |
| ENSMUSG00000048922 | cell division cycle associated 2(Cdca2) | Metabolism/Cell proliferation/Regulation |
| ENSMUSG00000048327 | cytoskeleton associated protein 2-like(Ckap2l) | Metabolism/Cell proliferation/Regulation |
| ENSMUSG00000047889 | serine (or cysteine) peptidase inhibitor, clade B, member 6d(Serpinb6d) | Metabolism/Cell proliferation/Regulation |
| ENSMUSG00000044951 | myosin light chain kinase family, member 4(Mylk4) | Metabolism/Cell proliferation/Regulation |
| ENSMUSG00000044201 | cell division cycle 25C(Cdc25c) | Metabolism/Cell proliferation/Regulation |
| ENSMUSG00000041431 | cyclin B1(Ccnb1) | Metabolism/Cell proliferation/Regulation |
| ENSMUSG00000037725 | cytoskeleton associated protein 2(Ckap2) | Metabolism/Cell proliferation/Regulation |
| ENSMUSG00000037628 | cyclin-dependent kinase inhibitor 3(Cdkn3) | Metabolism/Cell proliferation/Regulation |
| ENSMUSG00000037544 | discs, large (Drosophila) homolog-associated protein 5(Dlgap5) | Metabolism/Cell proliferation/Regulation |
| ENSMUSG00000035606 | kyphoscoliosis peptidase(Ky) | Metabolism/Cell proliferation/Regulation |
| ENSMUSG00000032218 | cyclin B2(Ccnb2) | Metabolism/Cell proliferation/Regulation |
| ENSMUSG00000030867 | polo-like kinase 1(Plk1) | Metabolism/Cell proliferation/Regulation |
| ENSMUSG00000028068 | IQ motif containing GTPase activating protein 3(Iqgap3) | Metabolism/Cell proliferation/Regulation |
| ENSMUSG00000027715 | cyclin A2(Ccna2) | Metabolism/Cell proliferation/Regulation |
| ENSMUSG00000027699 | ect2 oncogene(Ect2) | Metabolism/Cell proliferation/Regulation |
| ENSMUSG00000027379 | BUB1, mitotic checkpoint serine/threonine kinase(Bub1) | Metabolism/Cell proliferation/Regulation |
| ENSMUSG00000026683 | NUF2, NDC80 kinetochore complex component(Nuf2) | Metabolism/Cell proliferation/Regulation |
| ENSMUSG00000026416 | interleukin 20(Il20) | Metabolism/Cell proliferation/Regulation |
| ENSMUSG00000024791 | cell division cycle associated 5(Cdca5) | Metabolism/Cell proliferation/Regulation |
| ENSMUSG00000023505 | cell division cycle associated 3(Cdca3) | Metabolism/Cell proliferation/Regulation |
| ENSMUSG00000021965 | spindle and kinetochore associated complex subunit 3(Ska3) | Metabolism/Cell proliferation/Regulation |
| ENSMUSG00000021485 | Max dimerization protein 3(Mxd3) | Metabolism/Cell proliferation/Regulation |
| ENSMUSG00000019942 | cyclin-dependent kinase 1(Cdk1) | Metabolism/Cell proliferation/Regulation |
| ENSMUSG00000006398 | cell division cycle 20(Cdc20) | Metabolism/Cell proliferation/Regulation |
| ENSMUSG00000001403 | ubiquitin-conjugating enzyme E2C(Ube2c) | Metabolism/Cell proliferation/Regulation |
| ENSMUSG00000035683 | maternal embryonic leucine zipper kinase(Melk) | Lipid metabolism |
| ENSMUSG00000017716 | baculoviral IAP repeat-containing 5(Birc5) | Lipid metabolism |
| ENSMUSG00000099517 | histone cluster 1, H3g (Hist1h3g) | DNA repair |
| ENSMUSG00000078773 | RAD54 homolog B (S. cerevisiae)(Rad54b) | DNA repair |
| ENSMUSG00000069301 | histone cluster 1, H2ag(Hist1h2ag) | DNA repair |
| ENSMUSG00000041064 | PIF1 5'-to-3' DNA helicase(Pif1) | DNA repair |
| ENSMUSG00000039396 | nei like 3 (E. coli)(Neil3) | DNA repair |
| ENSMUSG00000035365 | PARP1 binding protein(Parpbp) | DNA repair |
| ENSMUSG00000001517 | forkhead box M1(Foxm1) | DNA repair |
| ENSMUSG00000032783 | trophinin associated protein(Troap) | Cell adhesion |
| ENSMUSG00000022033 | PDZ binding kinase(Pbk) | Immune Function |
| ENSMUSG00000074476 | SPC24, NDC80 kinetochore complex component, homolog (S. cerevisiae) (Spc24) | Function unknown |
| ENSMUSG00000040204 | RIKEN cDNA 2810417H13 gene(2810417H13Rik) | Function unknown |
| ENSMUSG00000036223 | spindle and kinetochore associated complex subunit 1(Ska1) | Function unknown |
| ENSMUSG00000034883 | leucine rich repeat protein 1(Lrr1) | Function unknown |
| ENSMUSG00000026955 | suppressor APC domain containing 2(Sapcd2) | Function unknown |
| ENSMUSG00000020808 | family with sequence similarity 64, member A(Fam64a) | Function unknown |
| ENSMUSG00000002055 | sperm associated antigen 5(Spag5) | Function unknown |
| ENSMUSG00000111243 | AC131777.1 | Function unknown |
| ENSMUSG00000108022 | Gm7298 | Function unknown |
| ENSMUSG00000102700 | Gm38312 | Function unknown |
| ENSMUSG00000100426 | Gm4208 | Function unknown |
| ENSMUSG00000097755 | 2010110K18Rik | Function unknown |
| ENSMUSG00000097484 | Gm26807 | Function unknown |
| ENSMUSG00000095026 | Gm3336 | Function unknown |
| ENSMUSG00000093726 | Gm20667 | Function unknown |
| ENSMUSG00000087067 | Gm11532 | Function unknown |
| ENSMUSG00000078505 | Gm436 | Function unknown |
| ENSMUSG00000034773 | BC030867 | Function unknown |

**Table S4. Detailed information of YNtreated nodes mapped in each cluster**

| Cluster | Node counts | YNtreated nodes mapped in each cluster | Percentage (%) |
| --- | --- | --- | --- |
| 1 | 266 | 145 | 54.51 |
| 2 | 270 | 197 | 72.96 |
| 3 | 279 | 133 | 47.67 |
| 4 | 15 | 15 | 100.00 |
| 5 | 55 | 19 | 34.55 |
| 6 | 137 | 129 | 94.16 |
| 7 | 35 | 28 | 80.00 |
| 8 | 19 | 5 | 26.32 |
| 9 | 18 | 15 | 83.33 |
| 10 | 3 | 1 | 33.33 |
| 11 | 7 | 0 | 0.00 |

**Table S5. The Top 20 nodes in Model net and YNtreated net.**

| No. | Hub nodes in each net (Top 20) | |
| --- | --- | --- |
|  | Model | YNtreated |
| 1 | MMP2 | |
| 2 | PLK1 | |
| 3 | CCNB1 | |
| 4 | TLR4 | |
| 5 | CDK1 | |
| 6 | CCNA2 | |
| 7 | CDC25C | |
| 8 | PDGFRA | |
| 9 | MYOC | |
| 10 | KNG1 | |
| 11 | IGFBP3 | LYN |
| 12 | BRCA1 | FN1 |
| 13 | TRIP13 | VAV1 |
| 14 | CHEK1 | PDGFRB |
| 15 | PRKG1 | BTK |
| 16 | BARD1 | CDC20 |
| 17 | MCM10 | ITGB3 |
| 18 | AURKA | GATA1 |
| 19 | RAD51 | MMP9 |
| 20 | CD5 | COL1A1 |

**Table S6. Primer sequences for Real-Time PCR.**

| Gene | direction | Primer pair sequence (5'→3') |
| --- | --- | --- |
| GAPDH | F  R | AGCAAGGACACTGAGCAAGA  GGGGTCTGGGATGGAAATTGT |
| MMP2 | F  R | CAAGTTCCCCGGCGATGTC  TTCTGGTCAAGGTCACCTGTC |
| TLR4 | F  R | ATGGCATGGCTTACACCACC  GAGGCCAATTTTGTCTCCACA |
| CDK1 | F  R | GGAAGGCCTGCTGAAGAGTT  TCCCTTAGCGCTTGTTCCTG |
| VAV1 | F  R | GGGGATCCACCACAACCATT  GTGTGGCACAGCAGTAGAG |

**The brief results of the analysis of DEGs in the inflamed skin samples relative to normal skin are listed below, which were published before.**

The DEGs between the control and inflamed skin samples were screened using *P*-value< 0.01 and |log_2_FC| > 3 as the thresholds. The top 100 upregulated DEGs were analyzed in detail. Thirty-eight of the top 100 upregulated genes encode immune response-related proteins, of which five (C7, C5ar1, F13a1, C6 and Cfh) are involved in complement and coagulation cascades, six (Serpina3n, Saa2, Saa1, Saa3, Lbp and Cd163) are related to the acute-phase response, five (Saa1, Saa2, Saa3, Cxcl13 and Ccl6) regulate chemotaxis, and four (Oas1a, Oas1f, Oas1g and Tlr8) are involved in virus-specific immune response. Twenty-one genes with the highest increase in transcript levels encode proteins that have known functions in metabolism and cell proliferation, whereas 8 are functionally uncharacterized. In addition, 14 upregulated genes encode structural proteins of the dermis, including tenascin N (Tnn), osteomodulin (Omd), cartilage intermediate layer protein (Clip), myocilin (Myoc), and multimerin 1 (Mmrn1). Three -encoding genes (Mmp8, Mmp11 and Lvrn) and seven genes encoding redox proteins also showed significant increase in their transcript levels.

Among the top 100 downregulated genes, 24 have unknown function. Only two genes encode immune response-related proteins, including PDZ binding kinase (Pbk) and transient receptor potential cation channel (Trpm1). Thirty-two genes regulate metabolism and cell proliferation, of which seven encode cell cycle proteins (Ccnb1, Cdk1, Ccnb2, Plk1, Cdc20, Cdc25c and Ccna2), and four encode negative regulators of cell proliferation (Hist1h2ag, Hist1h2ai, Nppc and Hist1h2an). The proteins encoded by Hist1h2ag and Hist1h2ai also play important roles in DNA repair. In addition, genes encoding DNA damage response proteins, including Exo1, Uhrf1, Ticrr, Kif22, Pclaf, Tgm6 and Pif1, were also significantly downregulated by *S. aureus* infection. Furthermore, 16 genes encode dermal proteins, of which 14 genes encode proteins related to keratin or keratin filament. Other major downregulated genes were those encoding proteins involved lipid metabolism (5) and transportation (10).

**GO and KEGG pathway analysis**

The top 5 GO terms for the up-regulated genes were regulation of response to stimulus, extracellular space, defense response, regulation of response to external stimulus and immune system process (shown in Fig. I. A), and that for the down-regulated DEGs were keratin filament, chromosome segregation, mitotic cell cycle process, cell cycle process and intermediate filament (shown in Fig. I. B). On the other hand, the most enriched KEGG pathways of the up-regulated genes were complement and coagulation cascades and *Staphylococcus aureus* infection (shown in Fig. I. C), and that of the down-regulated genes were cell cycle and oocyte meiosis (shown in Fig. I. D). GO clustering enrichment analysis further showed that the up-regulated DEGs were mainly involved in acute-phase response (enrichment score ES=3.06, gene count GC=8), chemotaxis (ES=2.86, GC=18), negative regulation of inflammation factor production (ES=2.12, GC=6) and lipid metabolism (ES=2, GC=10) (shown in Fig. II. A). The GO term of signal transduction was enriched in most up-regulated genes (39). As shown in Fig. II. B, the down-regulated DEGs were associated with cell cycle (ES=39.15, GC=70), chromosome segregation (ES=20.58, GC=59), structural molecule (ES=9.10, GC=22), microtubule-based movement (ES=8.72, GC=32), melanin biosynthetic process (ES=4.42, GC=7), and DNA metabolism (ES=2.21, GC=8).

**Figure I** The top 20 GO terms (A) and KEGG pathways (B) of different expressed genes enrichment in RNA-sequencing (*P*-value ≤ 0.05). Gene Counts: number of target genes in each GO term or KEGG pathway. Rich factor: the ratio of the number of target genes divided by the number of all the genes in each GO term or KEGG pathway. The size of pot indicated the gene counts, and the color reflected the different *P*-value.


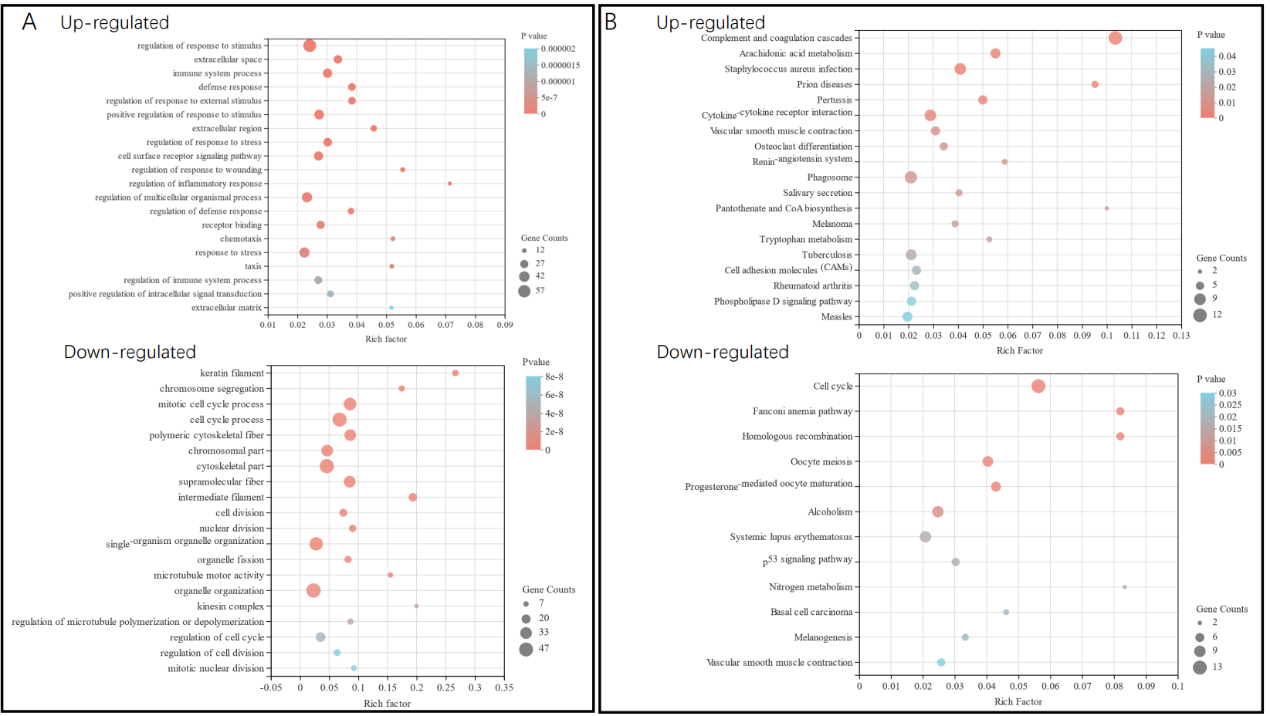


**Figure II** GO clustering results of DEGs with a criterion of Enrichment Score > 1. A, Up-regulated. B, Down-regulated. Counts represent the gene number enriched in each term. The Enrichment score was obtained according to the built-in program in DAVID, which ranks the biological significance of gene groups based on overall *P-*value of all enriched annotation terms. The higher the enrichment score is, the more important the term is.


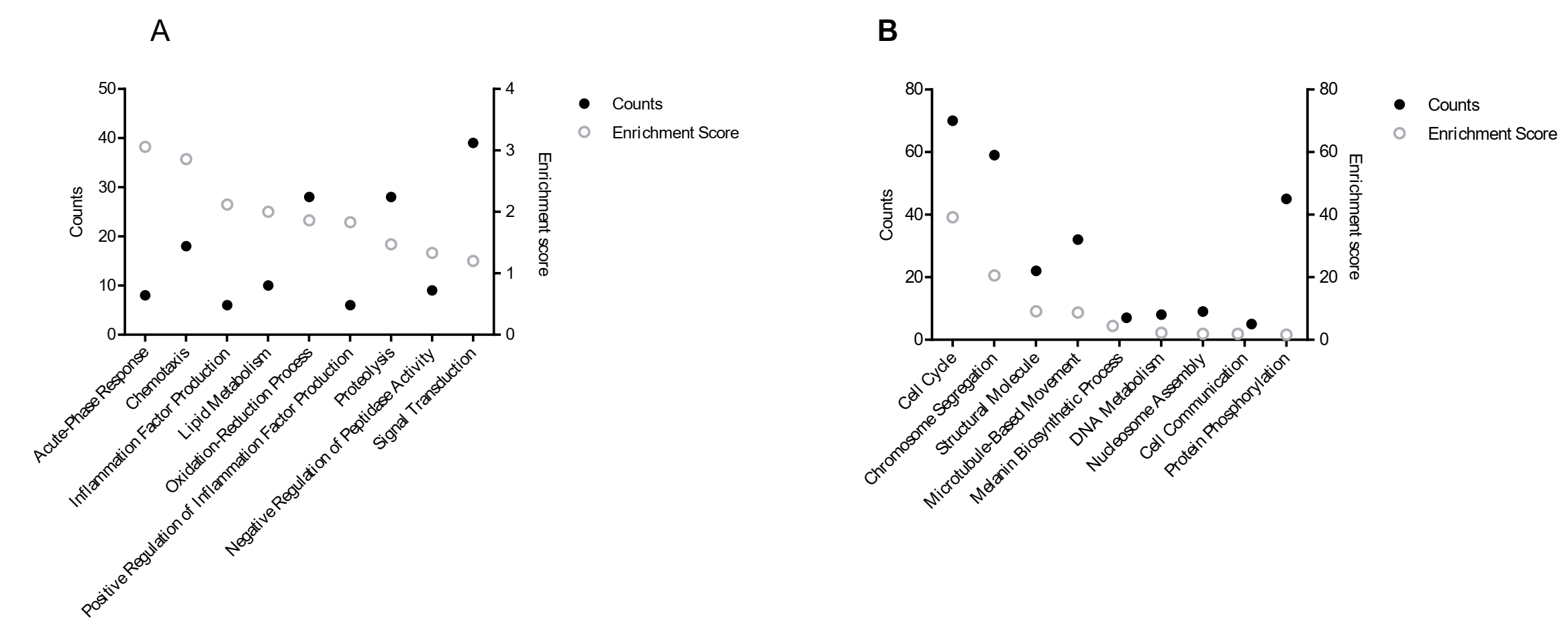


**PPI Network of DEGs and core genes in the PPI network**

The PPI network contained 1498 nodes (DEGs) and 6701 edges (interactions between the DEGs). Using the twofold median value of node degree in this network as a cutoff point, 1104 nodes were identified as hubs. Clustering analysis further revealed 11 groups, and the hub genes in each cluster were mainly associated with cell growth and death, cellular community, cell cycle, organismal systems, immune system, infectious diseases, signal transduction and apoptosis (shown in Table I). A total of 295 major hubs (degree higher than 2-fold of all hubs; median value=6) were screened and 4 clusters (naming Cluster 1 to 4) were built. As shown in Table II, the top 5 pathways in Cluster 1 were cell cycle (*P*- value = 1.17E-30), p53 signaling pathway (*P*-value = 1.76E-12), DNA replication (*P*-value = 7.53E-09), viral carcinogenesis (*P*-value = 3.20E-07), and homologous recombination (*P*-value = 2.31E-06), that in Cluster 2 were ErbB signaling pathway (*P*-value = 1.57E-18), Natural killer cell mediated cytotoxicity (*P*-value = 4.44E-17), PI3K-Akt signaling pathway (*P*-value = 2.55E-16), pathways in cancer (*P*-value = 8.16E-16) and proteoglycans in cancer (*P*-value = 3.22E-15), in Cluster 3 were pathways in cancer (*P*-value = 1.29E-27), chronic myeloid leukemia (*P*-value = 1.52E-21), hepatitis B (*P*-value = 1.13E-20), pancreatic cancer (*P*-value = 1.40E-19), and prostate cancer (*P*-value = 1.57E-19), and in Cluster 4 were oocyte meiosis (*P*-value = 0.005), legionellosis (*P*-value = 0.05), and salmonella infection (*P*-value = 0.08). Furthermore, the top 4 high-degree hub nodes in each cluster included CDK1, MCM10, CD5, PDGFRA, BRCA1, LEF1, AURKA, BUB1 etc. (shown in Table 5), and were the most down-regulated genes.

**Table I Main GO and pathway enrichment analysis relating to all hubs**

| Clusters in network | GO and pathway enrichment analysis |
| --- | --- |
| 1 | Cell growth and death |
| 2 | Cellular community |
| 3 | Infectious diseases and cancers |
| 4 | Cell cycle |
| 5 | Organismal Systems |
| 6 | Immune system |
| 7 | Infectious diseases |
| 8 | Signal transduction |
| 9 | Apoptosis |
| 10 | —— |
| 11 | —— |

Abbreviations: GO, Gene ontology.

**Table II Pathway enrichment analysis of PPI clusters related to major hubs (Top 5)**

| Clusters | Pathway enrichment analysis (KEGG)^‡^ | Count^†^ | *P*-value |
| --- | --- | --- | --- |
| 1 | hsa04110: Cell cycle | 26 | 1.17E-30 |
|  | hsa04115: p53 signaling pathway | 12 | 1.76E-12 |
|  | hsa03030: DNA replication | 8 | 7.53E-09 |
|  | hsa05203: Viral carcinogenesis | 12 | 3.20E-07 |
|  | hsa03440: Homologous recombination | 6 | 2.31E-06 |
| 2 | hsa04012: ErbB signaling pathway | 19 | 1.57E-18 |
|  | hsa04650: Natural killer cell mediated cytotoxicity | 20 | 4.44E-17 |
|  | hsa04151: PI3K-Akt signaling pathway | 28 | 2.55E-16 |
|  | hsa05200: Pathways in cancer | 29 | 8.16E-16 |
|  | hsa05205: Proteoglycans in cancer | 22 | 3.22E-15 |
| 3 | hsa05200: Pathways in cancer | 46 | 1.29E-27 |
|  | hsa05220: Chronic myeloid leukemia | 22 | 1.52E-21 |
|  | hsa05161: Hepatitis B | 27 | 1.13E-20 |
|  | hsa05212: Pancreatic cancer | 20 | 1.40E-19 |
|  | hsa05215: Prostate cancer | 22 | 1.57E-19 |
| 4 | hsa04114: Oocyte meiosis | 3 | 0.00514 |
|  | hsa05134: Legionellosis | 2 | 0.053695 |
|  | hsa05132: Salmonella infection | 2 | 0.081498 |

†: Count represents the gene number.

‡: KEGG, Kyoto Encyclopedia of Genes and Genomes.
